# Human longevity and Alzheimer’s disease variants act via microglia and oligodendrocyte gene networks

**DOI:** 10.1093/brain/awae339

**Published:** 2025-01-09

**Authors:** Andrew C Graham, Eftychia Bellou, Janet C Harwood, Umran Yaman, Meral Celikag, Naciye Magusali, Naiomi Rambarack, Juan A Botia, Carlo Sala Frigerio, John Hardy, Valentina Escott-Price, Dervis A Salih

**Affiliations:** UK Dementia Research Institute at University College London, London WC1E 6BT, UK; UK Dementia Research Institute at Cardiff University, School of Medicine, Cardiff University, Cardiff CF24 4HQ, UK; Division of Psychological Medicine and Clinical Neurosciences, School of Medicine, Cardiff University, Cardiff CF24 4HQ, UK; UK Dementia Research Institute at University College London, London WC1E 6BT, UK; UK Dementia Research Institute at University College London, London WC1E 6BT, UK; UK Dementia Research Institute at University College London, London WC1E 6BT, UK; UK Dementia Research Institute at University College London, London WC1E 6BT, UK; Communications Engineering and Information Department, University of Murcia, 30100, Murcia, Spain; Department of Neurodegenerative Diseases, Institute of Neurology, University College London, London WC1N 1PJ, UK; UK Dementia Research Institute at University College London, London WC1E 6BT, UK; UK Dementia Research Institute at University College London, London WC1E 6BT, UK; Department of Neurodegenerative Diseases, Institute of Neurology, University College London, London WC1N 1PJ, UK; UK Dementia Research Institute at Cardiff University, School of Medicine, Cardiff University, Cardiff CF24 4HQ, UK; Division of Psychological Medicine and Clinical Neurosciences, School of Medicine, Cardiff University, Cardiff CF24 4HQ, UK; UK Dementia Research Institute at University College London, London WC1E 6BT, UK

**Keywords:** Alzheimer’s disease, ageing, microglia, oligodendrocytes, myelin, homeostasis

## Abstract

Ageing underlies functional decline of the brain and is the primary risk factor for several neurodegenerative conditions, including Alzheimer’s disease (AD). However, the molecular mechanisms that cause functional decline of the brain during ageing, and how these contribute to AD pathogenesis, are not well understood.

The objective of this study was to identify biological processes that are altered during ageing in the hippocampus and that modify Ad risk and lifespan, and then to identify putative gene drivers of these programmes. We integrated common human genetic variation associated with human lifespan or Ad from genome-wide association studies with co-expression transcriptome networks altered with age in the mouse and human hippocampus.

Our work confirmed that genetic variation associated with Ad was enriched in gene networks expressed by microglia responding to ageing and revealed that they were also enriched in an oligodendrocytic gene network. Compellingly, longevity-associated genetic variation was enriched in a gene network expressed by homeostatic microglia whose expression declined with age. The genes driving this enrichment include *CASP8* and *STAT3*, highlighting a potential role for these longevity-associated genes in the homeostatic functions of innate immune cells, and these genes might drive ‘inflammageing’. Thus, we observed that gene variants contributing to ageing and AD balance different aspects of microglial and oligodendrocytic function. Furthermore, we also highlight putative Ad risk genes, such as *LAPTM5*, *ITGAM* and *LILRB4*, whose association with Ad falls below genome-wide significance but show strong co-expression with known Ad risk genes in these networks. Indeed, five of the putative risk genes highlighted by our analysis, *ANKH*, *GRN*, *PLEKHA1*, *SNX1* and *UNC5CL*, have subsequently been identified as genome-wide significant risk genes in a subsequent genome-wide association study with larger sample size, validating our analysis.

This work identifies new genes that influence ageing and AD pathogenesis, and highlights the importance of microglia and oligodendrocytes in the resilience of the brain against ageing and AD pathogenesis. Our findings have implications for developing markers indicating the physiological age of the brain and new targets for therapeutic intervention.

## Teaser

Graham *et al.* show that Alzheimer's disease-associated genetic variants are enriched in gene networks expressed by microglia and oligodendrocytes during ageing. By contrast, longevity-associated variants are enriched in gene networks expressed by homeostatic microglia, downregulation of which may contribute to ‘inflammageing’.

## Introduction

Biological ageing is the decline of adult tissue function following development. The mammalian CNS shows various impairments in function with age, such as impaired spatial memory, episodic memory and executive function.^1-3^ This decline in function has been linked to cellular changes in different brain regions, including modification of synaptic connections, and inflammatory changes.^3,4^ Ageing is considered the primary risk factor for a number of neurodegenerative conditions, including Alzheimer’s disease (AD); however, the molecular mechanisms linking ageing and AD are not well understood.^1,5,6^ Therefore, understanding the mechanisms of biological ageing might not only facilitate combatting the widespread and growing morbidity caused by age-related CNS decline, but also provide insights into the onset and progression of age-dependent diseases, such as AD.

Ageing affects all cell types in the brain, from neurons to the macrophages resident within the brain parenchyma, microglia and the supporting vasculature. Emerging data are also revealing a prominent disruption of myelin with ageing.^7-9^ Myelin is a layered lipid-rich membrane produced by oligodendrocytes that ensheathes neuronal axons, providing metabolic support and enabling fast propagation of action potentials. Myelin degrades with age, starting from ∼50 years of age in humans. This includes hyperintensities seen by MRI, in addition to myelin outfolding and accumulation of multilamellar fragments seen by electron microscopy. Axonal function can be restored via the proliferation and differentiation of oligodendrocyte precursor cells into mature oligodendrocytes that lay down new myelin at sites of lesions.^9,10^ However, remyelination becomes impaired in the aged brain. Microglia are thought to play an important protective role in the remyelination process by clearing degenerated myelin that accumulates during ageing and disease through phagocytosis and by secretion of growth factors and cytokines to support oligodendrocyte maturation and remodel the extracellular matrix.^8,9,11,12^ Recent work has shown that myelin disruption with ageing in rodents can drive accumulation of amyloid plaques by entangling microglia, potentially contributing to AD pathogenesis.^13^ Although these studies shed light on age-associated changes in the brain, it is not clear which of the many altered pathways are ultimately drivers of brain ageing, and hence which pathways should be targeted to prevent age-dependent CNS disorders.

Genome-wide association studies (GWAS) have provided invaluable insight into the causes of human disease. They have been used to identify genetic variation associated with human ageing and age-related diseases, with the identification of genes such as *APOE* and *FOXO3*, which are associated with ageing in some populations.^14,15^ Genetic variation in these genes might underlie some of the differences in ageing between people who are long-lived versus those that live an average lifespan. However, these genes are often multifunctional, and it is not clear which of their functions influence ageing and/or AD risk.

To gain a better understanding of the age-dependent processes within the CNS that contribute to lifespan and AD pathogenesis, we used a three-step approach to identify biological pathways that are dysregulated with age and are enriched for genes associated with longevity or AD risk. First, we identified common human genetic variation associated with ageing or AD at the gene-based level by aggregating the significance of individual single nucleotide polymorphisms (SNPs) and by testing the joint summary of all SNPs in the gene, accounting for linkage disequilibrium (LD) and the number of SNPs per gene. Second, we assessed the enrichment of associated genes in age-related gene co-expression networks, generated from bulk transcriptomic profiling of the human and mouse hippocampus, and single-cell RNA sequencing (scRNA-seq) of mouse hippocampal microglia. We found a statistically significant enrichment of genes harbouring collective SNP variants associated with AD risk within gene expression networks expressed by microglia and oligodendrocytes, which are upregulated during human hippocampal ageing. Furthermore, we found an enrichment of genes associated with ageing in humans in a network highly expressed by homeostatic microglia, which is downregulated with age and demyelination. Finally, we performed a transcriptome-wide association study (TWAS) to identify and confirm new risk genes associated with longevity, and we compared the results with the genes associated with AD in the recently published AD TWAS.^16^ Our data suggest a hypothesis whereby potentially linked microglial and oligodendrocytic responses to ageing might modify AD risk, whereas the degree of age-dependent loss of homeostatic microglial functions might influence longevity. These findings might allow earlier tracking of disease progression, provide new targets for AD prevention and facilitate further work into healthy CNS ageing.

## Materials and methods

### Sample cohorts

#### Alzheimer’s disease

We used the publicly available GWAS summary statistics data from the International Genomics of Alzheimer’s Projects (IGAP) from the stage 1 GWAS meta-analysis of 21 982 AD cases and 41 944 cognitively normal controls.^17^

#### Ageing

We used the publicly available summary statistics data from European-ancestry GWAS data with overlapping ageing traits, incorporating three GWASs covering healthspan (300 477 individuals, of whom 28.3% were not healthy), parental lifespan (1 012 240 parents, of whom 60% were deceased) and longevity (defined by 11 262 individuals surviving to the 90th percentile of life and 25 483 controls whose age at death corresponds to the 60th survival percentile).^14,15^

### Gene-based analysis

Gene-based testing was performed using MAGMA v.1.08^18^ to obtain gene-based significance (*P*-values) using GWAS summary statistics (details are provided in the Supplementary material). We ran the analyses with (35 kb upstream and 10 kb downstream as suggested by Wu *et al*.^19^) and without an annotation window around the genes. We report the gene-based *P*-values both before and after false discovery rate (FDR) correction for analyses with and without the annotation window.

### RNA-sequencing data pre-processing

Transcripts per million (TPM) normalized bulk hippocampal RNA-sequencing (RNA-seq) datasets were generated from wild-type C57BL/6J mice (8, 16, 32 and 72 weeks of age; data available from Mouseac.org)^20^ and non-diseased human hippocampi [from 196 individuals from the Genotype-Tissue Expression (GTEx) Project; v.8 TPM counts downloaded from gtexportal.org/home/datasets].^21^ Bulk microglial RNA-seq datasets were generated from microglia isolated from the hippocampus of aged and adult wild-type C57BL/6J mice^22^ or the corpus callosum of C57BL/6J mice treated for 0, 5 or 12 weeks with a demyelinating cuprizone diet.^23^ To assess module preservation, we used normalized bulk RNA-seq datasets generated from the hippocampi of wild-type mice of different ages.^24-26^

Raw counts generated by plate-based scRNA-seq of microglia isolated from the hippocampi of 3-, 6-, 12- and 21-month-old wild-type mice were downloaded from GSE127893; samples were removed if non-hippocampal or from *App* knock-in mice with Swedish (KM670/671NL), Arctic (E693G) and Beyreuther/Iberian (I716F) familial mutations.^27^ For details of data preprocessing, see the Supplementary material.

### Co-expression network analysis

Co-expression analysis was performed on pre-processed datasets using the *getDownstreamNetwork* function from the CoExpNets package.^28,29^ Modules formed by co-expression analysis were assessed for enrichment of cell type-specific genes using the *genAnnotationCellType* function of CoExpNets. Module enrichment for biological annotations was assessed using the *gost* function^30^ of the Gprofiler2 R package (for details, see the Supplementary material). Predicted annotations were excluded, and *P*-values were Bonferroni corrected. Module expression by age was assessed by Student’s two-tailed *t*-test of the mean expression of the 100 most central genes of the module (genes with highest correlation with the module eigengene) in the processed dataset of O’Neil *et al*.^22^ Module expression in cuprizone treatment groups was assessed by one-way ANOVA followed by the pairwise comparison of cuprizone diet time points to control diet using Dunnett’s test, if the ANOVA indicated a significant difference between treatment groups (*P* < 0.05), in the processed dataset of Nugent *et al*.^23^

Module preservation between our networks was calculated in the pre-processed datasets described above, using the *preservationoneway* function of CoExpNets. This returned preservation statistics calculated by the weighted gene co-expression network analysis (WGCNA) *modulePreservation* function.^31^ Comparison between human and mouse datasets was preceded by conversion of gene symbols to orthologues using the biomaRt R package.^32^ Module eigengenes were detected in query datasets using the *getNetworkEigengenes* function of CoExpNets. For further details, see Supplementary material.

### Pseudotime analysis

We used the Monocle 2 R package to infer single-cell transcriptional trajectories,^33,34^ based on the expression of genes significantly differentially expressed (FDR < 0.025) between cell clusters identified by Sala Frigerio *et al*.^27^

### Enrichment analyses of identified overlapping genes

Enrichment analyses were performed by comparing the number of overlapping statistically significant genes (gene-based *P* ≤ 0.01) showing association with AD or longevity derived from summary statistics from Kunkle *et al*.^17^ and Timmers *et al*.,^14^ respectively, in our transcriptome module gene sets with gene sets randomly bootstrapped (number of interations = 100 000). The enrichment analyses *P*-values (bootstrap *P*-values in the text) were calculated as the number of times when the random set of genes had larger or equal number of nominally significant genes in the module, divided by the total number of iterations. We report the bootstrap *P*-values based on the coordinates of genes without 35 kb upstream and 10 kb downstream window for two reasons: (i) to minimize the overlap between closely located genes attributable to their physical position; and (ii) to use the most conservative analysis, because the gene-based *P*-values were more conservative without the 35 and 10 kb flanking windows.

### Transcriptome-wide association study

Expression weights were used in TWAS for autosomal chromosomes, excluding the MHC region, with the longevity summary statistics,^14^ using the R script FUSION.assoc_test.R from the software FUSION.^35^ The TWAS weights were downloaded from the FUSION website [GTEX7 Brain (13 tissues) and GTEX7 Whole blood],^21,36^ Young Finns Study blood,^37^ Netherlands Twin Register blood,^38^ and for all monocytes^39^ as described by Harwood *et al*.^16^ Bonferroni correction for the number of genes analysed in each tissue was used to determine significance of TWAS associations. For further details, see the Supplementary material.

## Results

### Identification of genes harbouring common genetic variation associated with longevity and AD

Initially, we identified genes harbouring common genetic variants associated with lifespan to capture the collective SNPs of each gene, even if a single SNP does not reach genome-wide significance, by performing a gene-based analysis with the summary statistics from a meta-analysis combining three different GWAS associated with lifespan.^14^ We also performed the same gene-based analysis with an established GWAS of AD risk.^17^ The gene-based analysis identified 16 genes associated with lifespan (Supplementary Table 1) and 45 genes associated with AD at the gene-based genome-wide significance level (Supplementary Table 1) (*P* ≤ 1 × 10^−6^), corresponding to 7 and 12 loci, respectively (we assigned genes to the same locus, if they were in the ±500 kb region, as in the study by Bellenguez *et al*.^40^). The majority of the loci were previously identified via genome-wide significant SNPs in the original GWAS in the corresponding manuscripts.

As expected, the *APOE* locus was associated at the gene-based level to both GWAS (longevity, *P*_FDR_ = 1.95 × 10^−46^; AD, *P*_FDR_ = 1.99 ×10^−77^). We identified two additional loci containing *FURIN* and *FES* genes (CHR15:91411822–91439006, *P*_FDR_ = 9.8 × 10^−4^ and 2.06 × 10^−4^, respectively) and *KNOP1* and *IQCK* genes (CHR16:19713256–19869789, *P*_FDR_ = 3.3 × 10^−3^ and 3.9 × 10^−4^, respectively) associated with longevity and AD, respectively. These genes were not identified at the SNP genome-wide significance level by the GWAS summary statistics, which we used in this study. Both loci have, however, been identified in subsequent meta-analyses. There were 30 and 77 FDR-corrected significant (*P*_FDR_ ≤ 0.01) genes associated with lifespan and AD (Supplementary Table 1), corresponding to 17 and 25 loci.

### Transcriptional networks of oligodendrocytes and microglia are associated with age in hippocampus

**Figure 1:**
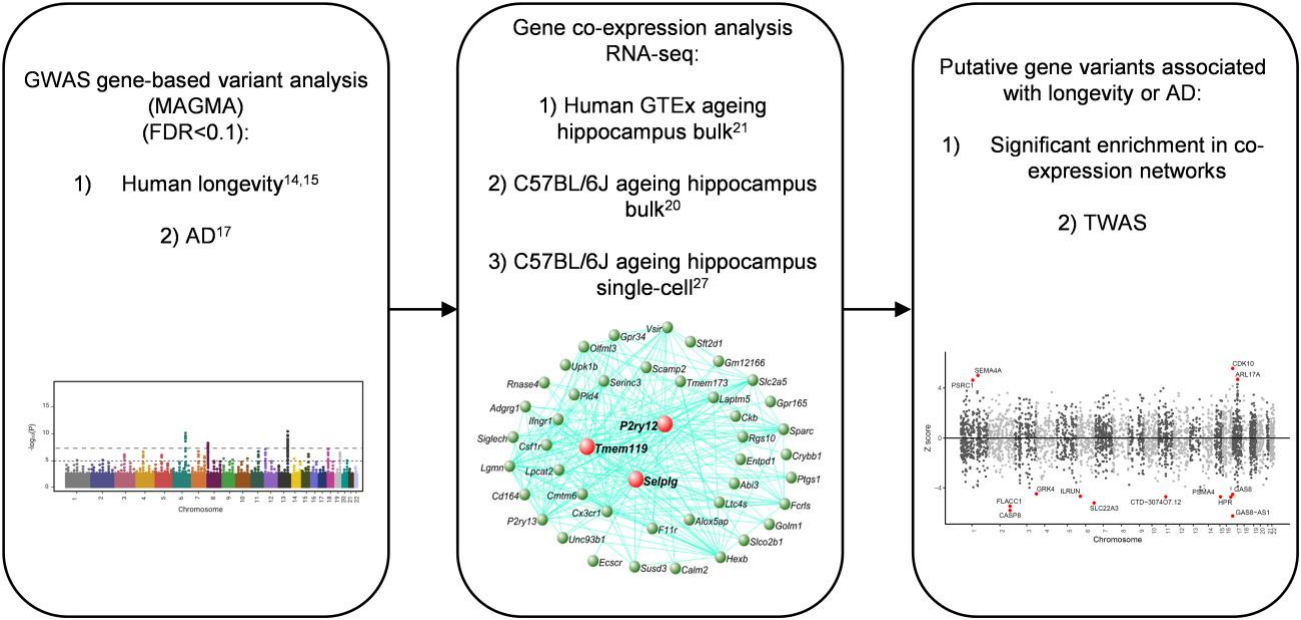
**Experimental workflow to integrate human gene variants associated with AD and longevity from GWAS with gene expression networks that show ageing-dependent changes.** Gene-based SNP analysis from GWAS for human longevity^14,15^ and AD^17^ (*left*). Gene co-expression networks that were ageing dependent were generated from RNA sequencing of bulk human hippocampus,^21^ bulk C57BL/6J mouse hippocampus^20^ and single-cell C57BL/6J mouse hippocampal microglia^27^ (*middle*). The gene-based GWAS were then used to identify enrichment of genes with variants in ageing gene co-expression networks or in TWAS from various expression datasets related to brain ageing^16^ (*right*). Details of the approach are in the ‘Materials and methods’ section and the Supplementary material. AD = Alzheimer’s disease; FDR = false discovery rate; GTEx = Genotype-Tissue Expression Project; GWAS = genome-wide association studies; MAGMA = multi-marker analysis of genomic annotation^18^; SNP = single nucleotide polymorphism; TWAS = transcriptome-wide association study.^16^

**Figure 2:**
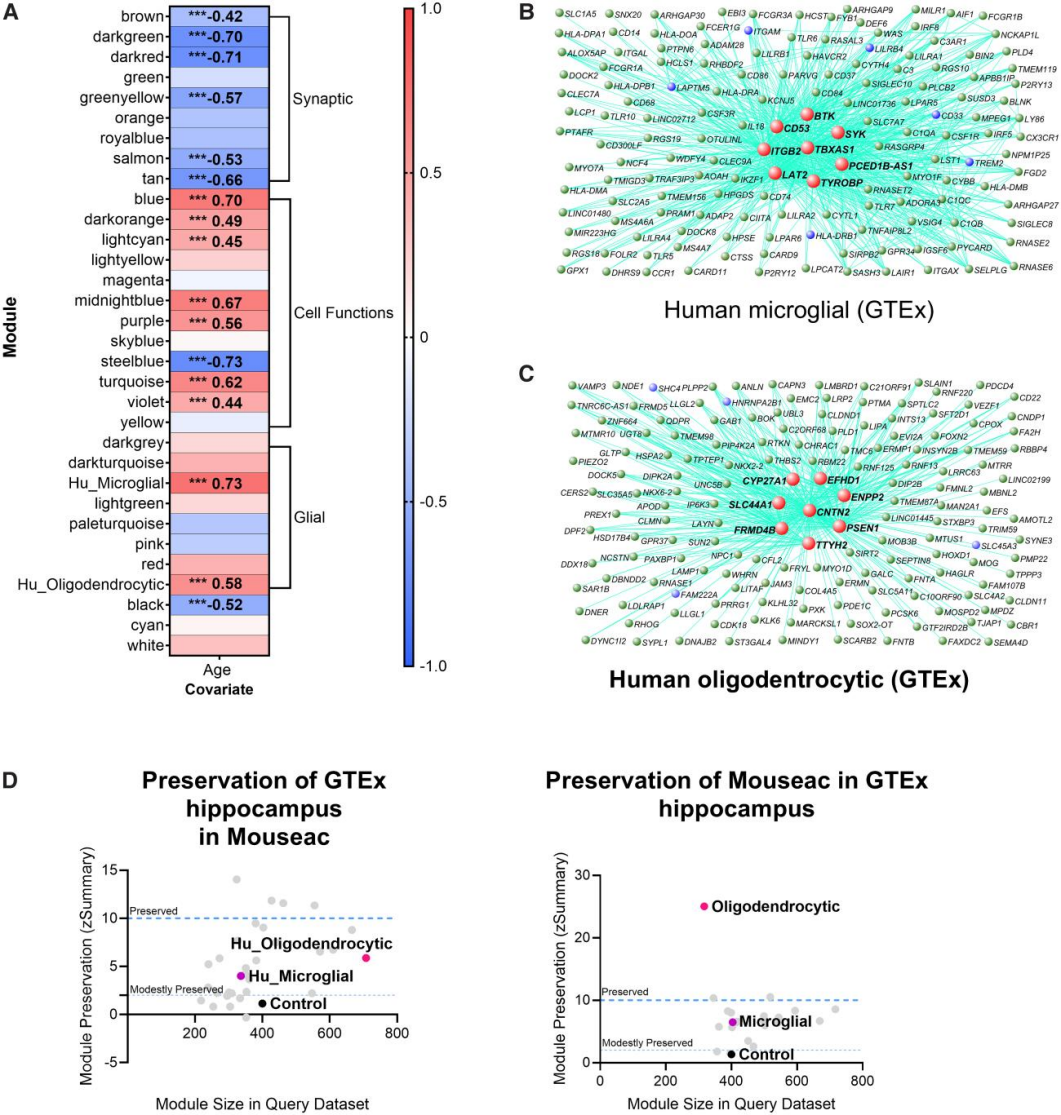
**Co-expression analysis of the human hippocampus reveals aspects of ageing and features preserved in mice or unique to humans.** (**A**) Co-expression analysis produced 17 gene modules whose expression is significantly correlated with age in the human hippocampus (correlation > 0.4), from the GTEx data.^21,36^ ***P < 0.001. (**B**) Network diagram of the most central 152 genes from the human microglial network. Red nodes represent hub genes, and blue nodes represent known and putative AD risk genes. (**C**) Network diagram of the most central 150 genes from the human oligodendrocytic network. (**D**) Mouse co-expression modules are, in general, moderately preserved in humans. Therefore, it appears that only subsections of the mouse gene networks are preserved in humans. Human GTEx co-expression module preservation in mouse data (*left*), and mouse co-expression module preservation in human GTEx RNA-sequencing data (*right*). z.summary is an amalgamation of other preservation statistics (z.density, mean connection strength per gene; and z.connectivity, sum of all connections) found to depict module preservation better than these statistics alone or simple gene overlap measures.^31^ Human data were compared with our Mouseac data.^20^ Full human networks given in Supplementary Table 2. Mouse networks given in Supplementary Figs 1 and 2, and Supplementary Table 3. GTEx = Genotype-Tissue Expression Project.

We then sought to identify which biological pathways contained these genes associated with longevity and AD from our analysis (Supplementary Table 1), while determining how these pathways are affected by ageing (experimental workflow in Fig. 1). To determine transcriptomic changes associated with human ageing at the systems level, we initially grouped genes that were co-expressed in the non-diseased hippocampi of 196 cognitively normal individuals from the GTEx consortium.^21^ We achieved this using WGCNA,^28^ with a *k*-means clustering optimization for constructing more biologically meaningful co-expression networks.^29^ This analysis produced several modules of co-expressed genes whose expression was significantly correlated with age (Fig. 2A). Strikingly, the module most positively correlated to age was enriched for microglial genes, which showed increased expression (Pearson’s product–moment correlation with age, here and hereafter, *R*^2^ = 0.73, *P* < 0.001; Hu_Microglial module), with genes such as *TYROBP*, *SYK* and *CD53* presenting as the most central or hub genes (Fig. 2A and B). Additionally, expression of an oligodendrocytic gene enriched module (*R*^2^ = 0.58, *P* < 0.001; Hu_Oligodendrocyte module) was also significantly positively associated with age, showing increased expression, with genes such as *ENPP2*, *CNTN2*, *PSEN1*, *SLC44A1* and *CYP27A1* presenting as hub genes (Fig. 2A and C).

To understand how the most common model of mammalian ageing recapitulates age-dependent expression changes in the human hippocampus, we also performed a similar analysis using RNA-seq data from the hippocampi of C57BL/6J wild-type mice at 2–18 months of age.^20^ As in humans, the module with strongest correlation with age (*R*^2^ = 0.93, *P* < 0.001) was heavily enriched for microglial genes (microglial module), which increased with ageing (Supplementary Figs 1 and 2). A second module that was significantly correlated with age (*R*^2^ = 0.54, *P* < 0.01) was heavily enriched for oligodendrocytic genes (mouse oligodendrocytic module), which increased with ageing. We saw strong preservation of both these modules, with respect to gene expression connectivity (z.summary co-expression patterns between module genes > 10) and correlation with age (microglial module *R*^2^ with age > 0.93 and oligodendrocytic module *R*^2^ > 0.58) in two other RNA-seq datasets of mouse hippocampal ageing (Supplementary Fig. 3). This indicates that these modules and their upregulation with age are preserved between different mice, laboratories and sequencing experiments.

Beyond these broad interspecies similarities, there were differences in the constituent genes and gene co-expression patterns of the human modules compared with their mouse counterparts. The human modules showed only moderate preservation of their gene co-expression patterns in the mouse data (Fig. 2D), and overlaps between gene orthologues in the human and mouse modules were modest but significant (microglial: 42 genes overlapped, *P* = 3.4 × 10^−26^, Fisher’s exact test; oligodendrocytic: 90 genes overlapped, *P* = 1.6 × 10^−75^, Fisher’s exact test). Age-dependent genes unique to humans were enriched for terms such as leucocyte activation, cytokine production and T-cell activation (Supplementary Fig. 4). Therefore, ageing of the human and mouse hippocampus is associated with an upregulation of networks of microglial and oligodendrocytic genes. Although these responses show core similarities, there are also significant interspecies differences.

### Human microglial and oligodendrocytic gene networks are enriched for AD risk genes

To investigate whether our age-dependent co-expression networks might be linked to AD risk, we tested the human and mouse age-dependent bulk RNA-seq co-expression networks for enrichment of genes, or mouse orthologues of human genes, significantly associated with AD (top genes associated with AD are given in Supplementary Table 1; *P*_FDR_ < 0.01). Enrichment analyses were performed by comparing the number of genes associated with Ad in our gene sets with randomly sampled gene sets matched for LD, gene length and the number of genes in a set. We saw a significant enrichment of genes associated with AD in both the age-dependent human microglial network (enrichment *P* = 1 × 10^−5^, bootstrap-based test) and oligodendrocytic network (enrichment *P* = 0.033, bootstrap-based test) (Fig. 2B and C and Table 1). In contrast, we saw no significant enrichment of common human AD-associated genes in the age-dependent mouse networks from bulk RNA-seq (Supplementary Fig. 1 and Table 1).

**Table 1:** Test of enrichment of age-dependent gene modules with genes variants associated with Alzheimer’s disease^17^ and longevity^14^ genome-wide association studies

	Alzheimer’s disease from GWAS of Kunkle *et al*.^17^			Ageing from GWAS of Timmers *et al*.^14^	
Module	No. of genes (Ad/ageing)	MAGMA *P*-value	Bootstrap *P*-value	MAGMA *P*-value	Bootstrap *P*-value
Human bulk microglial	434/423	0.023	**1 × 10^−5^**	0.982	0.806
Human bulk oligodendrocytic	780/764	0.406	**0**.**033**	0.871	0.804
Mouse bulk microglial	240/238	0.064	0.160	0.507	0.671
Mouse bulk ribosomal	333/321	0.429	0.515	0.510	0.472
Mouse bulk mitochondrial	396/389	0.711	0.975	0.163	0.801
Mouse bulk oligodendrocytic	236/235	0.989	0.943	0.776	0.638
Mouse single-cell interferon	308/305	0.045	0.280	0.284	0.553
Mouse single-cell ARM	499/487	0.503	0.067	0.721	0.969
Mouse single-cell phagolysosomal	1337/1310	0.608	0.054	0.759	0.146
Mouse single-cell HM2 homeostatic	964/936	0.533	0.329	0.734	**0**.**032**

**Figure 3:**
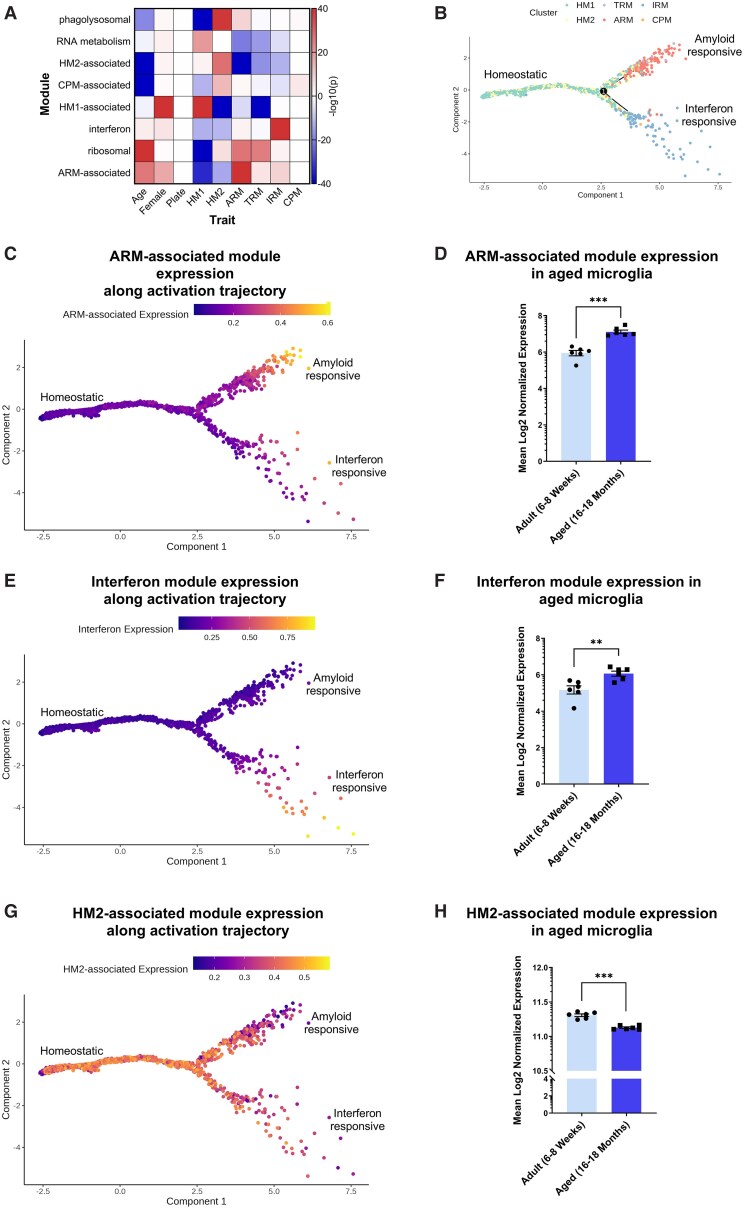
**Gene co-expression modules expressed by distinct microglial subpopulations are dysregulated with age in the mouse hippocampus.** (**A**) Association, *P*(−log10), between the expression of co-expression modules formed by co-expression analysis of data generated by single-cell RNA sequencing of microglia (Mg) isolated from wild-type mice at 3, 6, 12 and 21 months of age, and female sex, sequencing plate and six microglial subpopulations identified by the original authors.^27^ Subpopulations: HM1 = homeostatic microglia 1; HM2 = homeostatic microglia 2; TRM = transiting microglia; ARM = activated response microglia; IRM = interferon responsive microglia. Negative *P*(−log10) indicates a negative association. (**B**) Pseudotime analysis with Monocle 2 identified a bifurcating trajectory, with HM1 as a root state and with ARM and IRM as two terminal states. (**C**, **E** and **G**) Expression of the differentially expressed genes in the ARM-associated (**C**), interferon (**E**) and HM2-associated (**G**) co-expression modules shown along this Pseudotime trajectory. (**D**, **F** and **H**) Comparison of expression of the 100 most central genes (as a proxy for module expression) from the ARM-associated (**D**), interferon (**F**) and HM2-associated (**H**) modules in microglia isolated from the mouse hippocampus at 6–8 and 16–18 months of age and profiled by RNA sequencing.^22^  **D**, **F** and **H:**  *n* = 6 mice per age group, Student's *t*-test; *P < 0.05, **P < 0.01, ***P < 0.001. ARM = activated response microglia; HM2 = homeostatic microglial subcluster 2.

**Figure 4:**
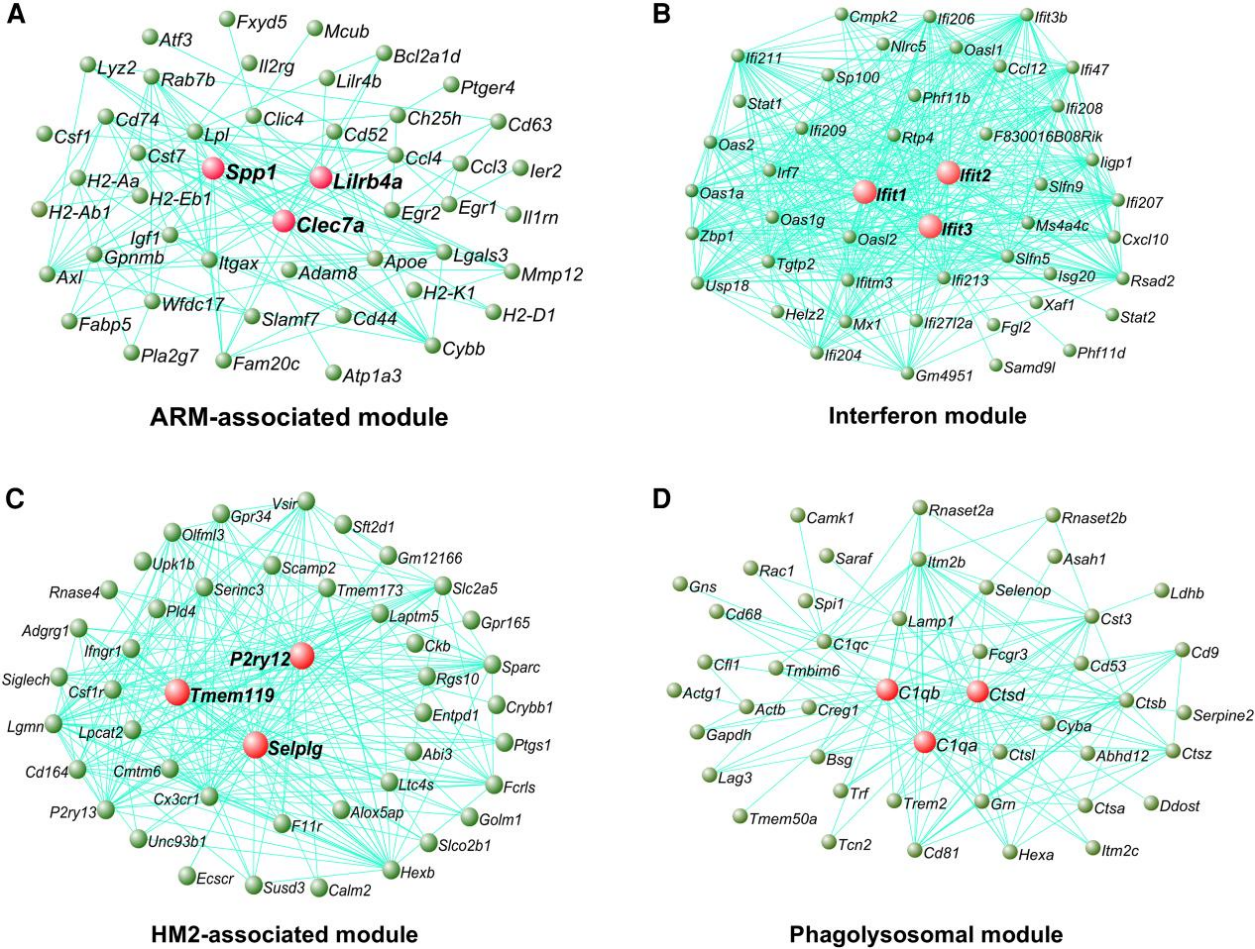
**Gene co-expression networks from microglia analysed by single-cell RNA sequencing associated with ageing.** The genetic networks from microglial cells isolated from wild-type mice at 3, 6, 12 and 21 months of age analysed by single-cell RNA sequencing.^27^ Network plot of the 45 most connected genes in the ARM-associated (**A**), interferon (**B**), HM2-associated (**C**) and phagolysosomal (**D**) modules. Green nodes represent genes, edge lines represent co-expression connections, and the central large red nodes are the hub genes. Full networks are given in Supplementary Table 6. ARM = activated response microglia; HM2 = homeostatic microglial subcluster 2.

Gene enrichment tests with MAGMA and bootstrap approaches. In the bootstrap approach, the number of genes with gene-based association *P* < 0.01 in the module is compared with the number of genes with *P* < 0.01 from a random gene set with the same number of genes. Gene modules from mouse hippocampus bulk RNA sequencing (Supplementary Fig. 1), mouse hippocampus single-cell RNA sequencing (Figs 3 and 4) and human hippocampus bulk RNA sequencing (Fig. 2). ARM = activated response microglia; GWAS = genome-wide association studies; HM2 = homeostatic microglial subcluster 2; MAGMA = multi-marker analysis of genomic annotation.^18^*P* < 0.05 (in bold).

The genes driving the enrichment (gene-based *P*-values ≤ 0.01) of the microglial module are known AD-associated risk genes (*CD33*, *GAL3ST4*, *HLA-DRB1*, *INPP5D*, *MS4A4A*, *SPI1* and *TREM2*) and putative risk genes (*ITGAM*, *LAPTM5* and *LILRB4*)^20,41^ (Fig. 2 and Supplementary Table 1). We also observed genes with gene-based *P*-values ≤ 0.01 not before linked to AD, which are likely to contribute to AD risk given their co-expression with other genes enriched for AD risk, namely, *APOC2*, *ARHGAP45*, *ATP8B4*, *CMTM7*, *COX7A1*, *DOK3*, *MARCO*, *NOP2*, *PCED1B* and *TMC8* (Table 1 and Supplementary Tables 1 and 4). Therefore, the age-dependent microglial genetic network identified here both highlights a role for microglia-associated age-related changes in determining AD risk and predicts new putative risk genes for AD.

None of the genes within the human oligodendrocytic module had previously been identified as GWAS hits for AD risk. Nevertheless, the gene *CLASRP* meets genome-wide significance in our analysis (*P*_FDR_ = 1.50 × 10^−10^), highlighting the benefit of our gene-based association analysis and indicating a potential oligodendrocytic role for this gene in the human hippocampus. *CLASRP* is in the same region as *APOE* but is included here as a gene of interest because it shows age-dependent expression changes. Moreover, the large number of co-expressed genes (29 genes) containing genetic variation associated with AD below our threshold (gene-based *P* ≤ 0.01; Table 1 and Supplementary Table 5) indicates that genetic variation that influences oligodendrocyte function during ageing might play a role in AD pathogenesis. Overall, our analysis suggests that microglial and oligodendrocytic responses during ageing might influence AD risk. Furthermore, many of the genes driving this influence might not be part of similar responses observed during ageing of the mouse hippocampus.

### Single-cell RNA-seq changes of age-dependent gene expression

Given that recent scRNA-seq studies have revealed that microglia respond heterogeneously to various challenges, we sought to determine age-dependent co-expression networks at the single-cell level, using scRNA-seq data generated from microglia isolated from male and female wild-type mouse hippocampi at 3, 6, 12 and 21 months of age (two mice of each sex/age, pooled prior to sequencing; data from Sala Frigerio *et al*.^27^). We used mouse rather than human data owing to the lack of scRNA-seq data covering human hippocampal microglia over the human lifespan and the difficulty in identifying the effect of age from the various environmental, technical and biological factors that affect gene expression and co-expression in human microglial scRNA-seq data.^42^

Previous analysis of these data identified six microglial subpopulations: homeostatic microglia 1 and 2 (HM1 and HM2), which highly express genes associated with microglial homeostatic functions; cycling proliferating microglia (CPM), which express genes involved in the cell cycle; activated response microglia (ARM); interferon-responsive microglia (IRM); and transiting microglia (TRM), which might be an intermediate state between homeostatic microglia and ARM.^27^ We identified eight co-expression modules expressed by distinct microglial sub-populations in the mouse hippocampus (Fig. 3A). To assess the age-related changes in expression of these modules reliably in more replicates than those available in scRNA-seq data, we assessed dysregulation of these modules (using the mean expression of the core 100 genes with the highest connectivity) between independent replicates of microglia isolated from wild-type mouse hippocampi at 2 and 16–18 months of age and profiled by bulk RNA-seq (data from O’Neil *et al*.^22^).

Six gene modules exhibited altered expression with age. A module of genes representing activated microglia exhibited strikingly increased expression with age (ARM-associated module, *P* = 1.2 × 10^−3^, Student’s *t*-test; Fig. 3A–D). This module was expressed predominantly by the ARM subpopulation of activated microglia, which are synonymous with the disease-associated microglia (DAM) subpopulations seen in AD mouse models.^27,43^ Interestingly, the hub genes of this module (*Spp1*, *Clec7a* and *Lilrb4a*; Fig. 4A) have been reported to mark microglia scavenging fragmented myelin in the corpus callosum, which also become more numerous with ageing.^8^ This module is significantly enriched for biological annotations related to phagocytosis, cytokine production and TYROBP signalling (Supplementary Fig. 5A). Another gene module consisting of interferon-stimulated genes was also increased with ageing (interferon module, *P* = 8.0 × 10^−3^, Student’s *t*-test; Figs 3E, F and 4B) and was associated with a distinct subpopulation of interferon-responsive microglia (IRM), hence it was named the interferon module. This module is significantly enriched for biological annotations related to the interferon response (Supplementary Fig. 5B). A further module containing ribosomal genes was more modestly increased with ageing (ribosomal module; *P* = 3.0 × 10^−2^, Student’s *t*-test; Supplementary Figs 5 and 6). In contrast, a module strongly associated with a homeostatic subcluster, HM2, showed decreased expression during ageing (HM2-associated module, *P* = 1.4 × 10^−5^, Student’s *t*-test; Figs 3G, H and 4C), while a module associated with a different homeostatic subpopulation of microglia, HM1 (HM1-associated module, *P* = 8.7 × 10^−3^, Student’s *t*-test) and a module associated with TGF-β signalling (TGF-β module, *P* = 4.0 × 10^−4^, Student’s *t*-test) were more modestly decreased during ageing (Supplementary Figs 5 and 6). Finally, modules containing phagocytosis and lysosomal genes (phagolysosomal module; Fig. 4D) or associated with the CPM cluster (CPM-associated module) were not significantly altered during ageing (*P* > 0.05, Student’s *t*-test).

### Single-cell RNA-seq signatures of microglia associated with ageing resemble the changes seen with demyelination

**Figure 5:**
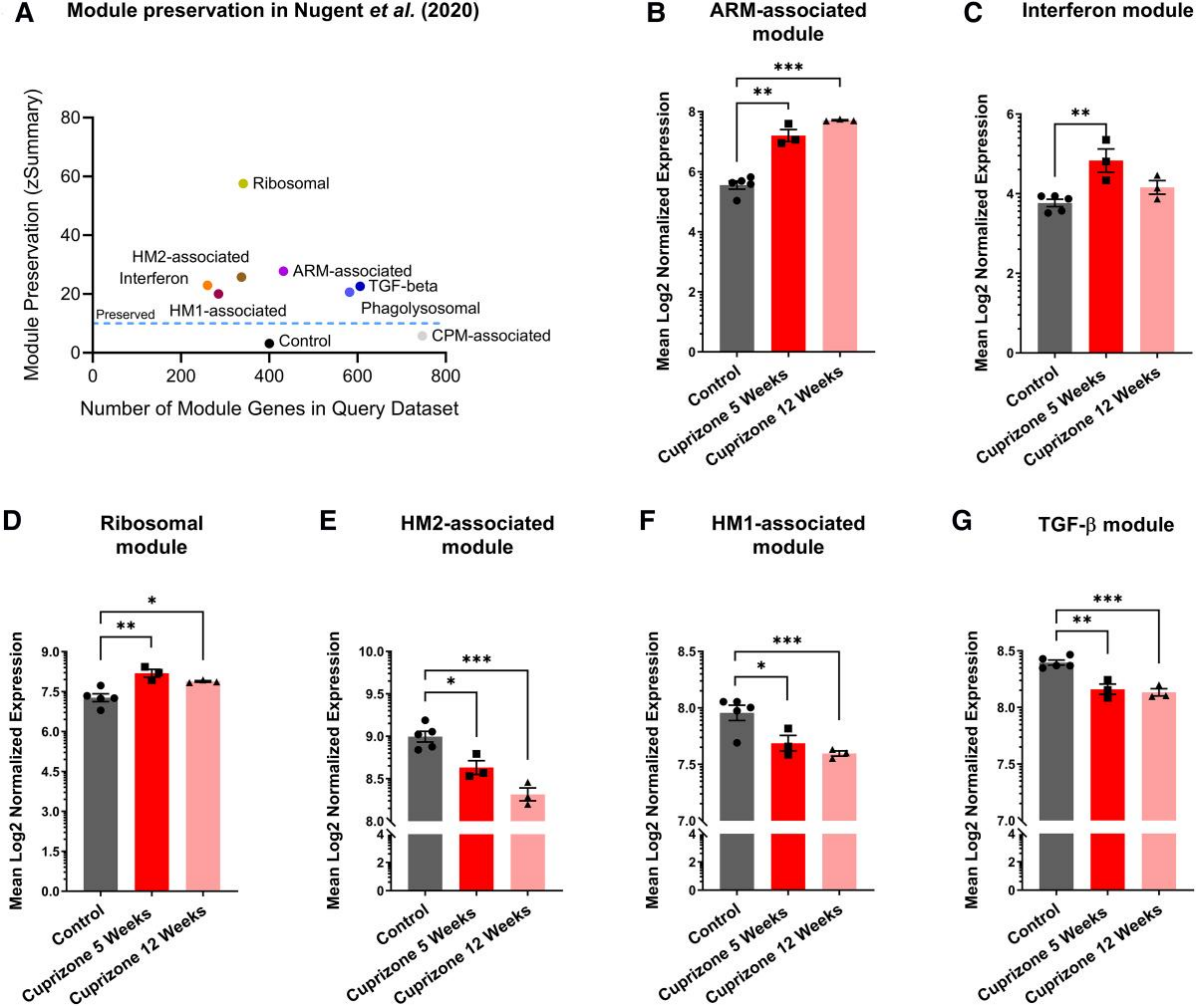
**Gene co-expression network changes in microglia isolated from mice treated with cuprizone and analysed using single-cell RNA sequencing.** (**A**) Preservation analysis of age-related modules in a dataset generated by single-cell RNA sequencing of microglia isolated from the corpus callosum of 9- to 11-month-old WT mice fed a control or demyelinating cuprizone diet for 5 or 12 weeks.^23^ Control module represents results for randomly chosen genes of a similar module size. (**B–G**) Comparison of expression of the 100 most central genes (as a proxy of module expression) from the ARM-associated (**B**), interferon (**C**), ribosomal (**D**), HM2-associated (**E**), HM1-associated (**F**) and TGF-β (**G**) modules in microglia isolated from the corpus callosum fed a control diet or a demyelinating cuprizone diet for 5 or 12 weeks and profiled by RNA sequencing.^23^  **B**–**G:**  *n* = 3–5 mice per diet group, module expression changes between cuprizone treatment groups was assessed by one-way ANOVA, if the ANOVA indicated a significant difference between treatment groups, a pairwise comparison of cuprizone diet timepoints to control diet was performed using Dunnett’s test; *P < 0.05, **P < 0.01, ***P < 0.001. ARM = activated response microglia; HM1 = homeostatic microglial subcluster 1; HM2 = homeostatic microglial subcluster 2.

We then sought to gain further insight into whether the microglial responses during ageing seen in our single-cell analysis might be influenced by interactions with oligodendrocytes, the other major source of age-related changes we identified in bulk RNA-seq data. Demyelination is a major feature of oligodendrocyte ageing in the mouse hippocampus.^44^ Therefore, we examined how these age-dependent scRNA-seq modules were affected when microglia were exposed to demyelination in young C57BL/6J mice, after feeding with cuprizone (data from Nugent *et al*.^23^). Interestingly, we found that the co-expression of genes within our age-associated microglial modules (Figs 3 and 4) was strongly preserved in young mice fed a demyelinating diet (Fig. 5A), indicating that these genes were also co-expressed in these mice exhibiting demyelination. Looking again at our age-dependent modules of interest (Figs 3 and 4), we found that the average expression of their 100 most connected genes showed the same change with demyelination as they showed with ageing. The ARM-associated module (ARM population) was increased after both 5 weeks (*P* = 6.7 × 10^−5^, Dunnett’s test) and 12 weeks (*P* = 9.3 × 10^−6^, Dunnett’s test) of a demyelinating cuprizone diet, mirroring the changes seen with ageing (Fig. 5B). The average expression of core interferon-response module genes was increased after 5 weeks (*P* = 4.0 × 10^−3^, Dunnett’s test) of a demyelinating cuprizone diet, as we saw during ageing, although this subsided after 12 weeks of the cuprizone diet (*P* = 0.24, Dunnett’s test; Fig. 5C), while the ribosomal module was more modestly elevated following 5 and 12 weeks on the cuprizone diet (5 weeks, *P* = 2.9 × 10^−3^; 12 weeks, *P* = 2.6 × 10^−2^; Dunnett’s tests; Fig. 5D). In contrast, the average expressions of core genes in the HM2-associated, HM1-associated and TGF-β modules were decreased after both 5 weeks (HM2, *P* = 1.3 × 10^−2^; HM1, *P* = 3.4 × 10^−2^; TGF-β, *P* = 1.2 × 10^−3^; Dunnett’s tests) and 12 weeks (HM2, *P* = 2.9 × 10^−4^; HM1, *P* = 7.8 × 10^−3^; TGF-β, *P* = 6.0 × 10^−4^; Dunnett’s tests) (Fig. 5E–G) of a demyelinating cuprizone diet, mirroring the repression of these modules during ageing (Fig. 3). Overall, these data provide evidence that the transcriptional programmes we identified were changed in a similar manner in microglia during ageing and in response to the breakdown of myelin in young mice.

### A homeostatic microglial gene network is enriched with common gene variants associated with longevity

To determine which specific age-dependent microglial transcriptional programmes are particularly associated with AD and longevity, we repeated our assessment of enrichment for common genetic variation associated with these traits, using modules from our scRNA-seq analysis (Fig. 3). Comparing the number of overlapping genes from our modules with bootstrapped modules containing the same number of genes randomly selected from those reliably detected from this dataset (to account for the relative enrichment of microglia-expressed genes with these traits), we observed a trend for enrichment of genes associated with AD within the genes present in the ARM- and phagolysosomal-associated modules (Figs 3 and 4, Table 1 and Supplementary Tables 6 and 7). Furthermore, our analysis suggests that the HM2-associated module is significantly, but modestly, enriched for genetic variation associated with longevity (enrichment *P* = 0.032, bootstrap-based test) (Table 1 and Supplementary Table 7). This homeostatic microglial module includes the mouse orthologue of *CASP8*, which acts as a molecular switch to control cell death via apoptosis, necroptosis and pyroptosis.^45^

### New genes associated with AD risk and genes of homeostatic microglia are associated with longevity

**Figure 6:**
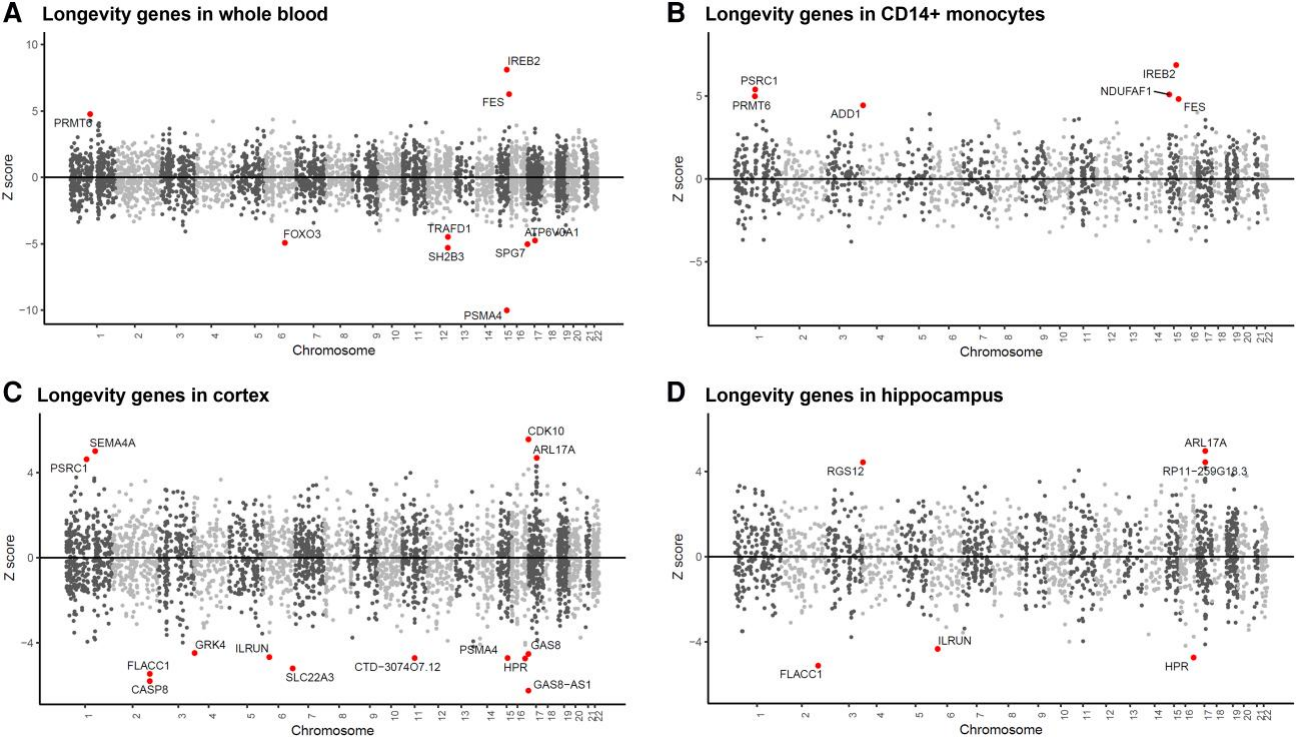
**Manhattan plots of TWAS results identifying genes whose expression is associated with longevity in whole blood, CD14^+^ myeloid cells, cortex and hippocampus.** The *y*-axis is the *Z*-score of the association between gene expression and longevity in (**A**) whole blood from Young Finns Study^37^; (**B**) naïve (CD14) monocytes from the Fairfax dataset^39^; (**C**) brain cortex from the GTEx Project^21,36^; and (**D**) hippocampus from the GTEx Project.^21,36^ Genes that showed significant association following Bonferroni correction for multiple testing are labelled (ageing risk genes, red).

**Figure 7:**
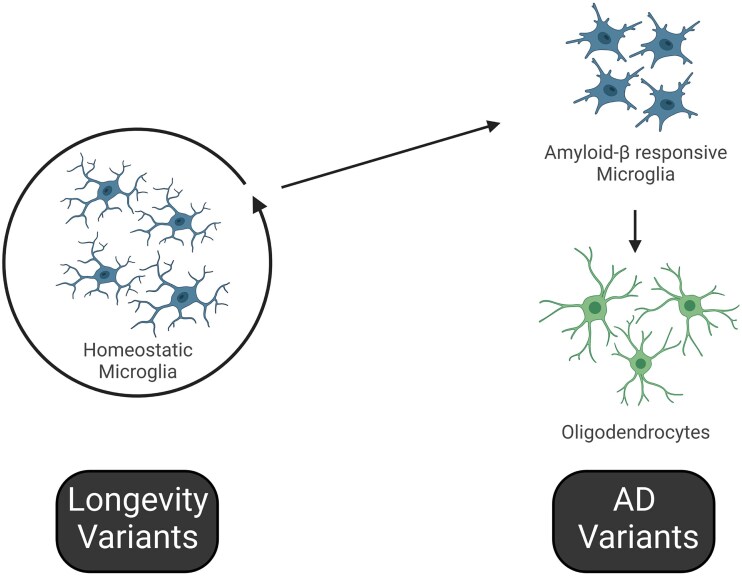
**Hypothesis for regulation of homeostatic microglia by genes associated with longevity, and on the other side regulation of microglial activity and oligodendrocyte function by genes associated with Alzheimer’s disease risk.** Our data suggest a model whereby genes associated with longevity are involved in the homeostatic functions of microglia and perhaps other innate immune cells, and that if these immune cells escape this state of homeostasis by activating stimuli such as amyloid pathology and age-dependent myelin fragmentation, then genes associated with Alzheimer's disease (AD) determine how microglia are activated and how microglia interact with remyelinating oligodendrocytes. The age-associated genes we identified in this study that putatively drive the genetic networks associated with innate immune homeostatic processes and activation might underlie ‘inflammageing’.

To investigate genetic variation associated with longevity using an independent approach, we performed a TWAS to identify genes in which a change in expression is associated with a genetic contribution to longevity. Data are not available to perform this analysis with microglia, but given that peripheral monocytes differentiate into microglia-like macrophages within the CNS, we used expression data from naïve (CD14) and induced monocytes (lipopolysaccharide and interferon-γ) from the Fairfax dataset,^39^ as previously performed for AD.^16^ In addition, we used expression data from the GTEx project, including the brain and hippocampus,^21,36^ whole blood from the Young Finns Study^37^ and the Netherlands Twin Register peripheral blood.^38^ These datasets include sorted CD14^+^ myeloid cells, whole blood and hippocampus, which can shed light on myeloid cell transcription. Using the AD TWAS, this approach confirmed the importance of known AD risk genes, such as *APOE*, *BIN1*, *CD33*, *CR1*, *MS4A4A*, *OAS1* and *SPI1* (extracted from the supplementary data of Harwood *et al*.^16^ and summarized in Supplementary Table 8). Furthermore, this approach also identified new risk genes not previously associated with AD, such as *GEMIN7* (frontal cortex, *P* = 2.17 × 10^−11^, *Z* = 6.69) and *MTCH2* (frontal cortex, *P* = 2.44 × 10^−10^, *Z* = −6.33) (extracted from the supplementary data of Harwood *et al*.^16^ and summarized in Supplementary Table 8). *GEMIN7* is in the same region as *APOE* but is included here as a gene of interest because it shows transcription-dependent association. Using the longevity GWAS, we noted known genes showing variation associated with longevity, including *FOXO3*, where reduced *FOXO3* expression was significantly associated with longevity in whole blood (Fig. 6). Additionally, we identified several genes associated with altered expression and longevity, such as *ADD1*, a cytoskeletal protein that achieved genome-wide significance in the CD14^+^ monocyte cell dataset, with increased expression being linked to longevity (Fig. 6). *CASP8* also achieved genome-wide significance in cortical tissue (frontal cortex, *P* = 3.71 × 10^−8^, *Z* = −5.50; Fig. 6 and Supplementary Table 8) and blood, with decreased expression being linked to longevity (Supplementary Fig. 7). Cathepsin S (*CTSS*) was also significant in the putamen, with increased expression being linked to longevity (Supplementary Fig. 7 and Supplementary Table 8). This approach has highlighted new genes associated with longevity (*P* ≤ 0.01 and *Z* > 3.0 or *Z* < −3.0), of which around half are thought to interact via ingenuity pathway analysis, including *APOE*, *CASP8*, *CTSB*, *CTSH*, *CTSS*, *LIPA*, *LPL*, *NPC1* and *TUFM* (Supplementary Fig. 8 and Supplementary Table 8). Importantly, *FOXO3*, *ADD1* and *CASP8* are expressed by homeostatic microglia. This supports our findings that genes associated with longevity are involved in the homeostatic functions of microglia and, perhaps, other innate immune cells (Fig. 7).

## Discussion

### Genetic variation associated with AD is enriched in age-dependent oligodendrocytes and microglia

A clear clue that links ageing and AD is that traditional GWAS have repeatedly demonstrated that *APOE* is the top genetic risk factor contributing to both late-onset AD^17^ and longevity,^14,15^ with the ɛ4 isoform being detrimental in both contexts, showing the highest risk for AD (with earlier age of onset among the late-onset AD cases)^46^ and the lowest odds for longevity. Additionally, longevity shows a significant negative genetic correlation with AD.^14^ However, the molecular mechanisms linking AD and ageing are poorly understood. To gain new insights into mechanisms linking ageing and AD, we have combined genetic association in human GWAS data with transcriptomic analyses of ageing in the human and mouse hippocampus, aiming to identify biological pathways and cell types that are both altered during ageing and associated with longevity and/or AD risk. We show that the expression of separate gene modules associated with microglia and oligodendrocytes exhibit a strong positive correlation with age in both species. However, the specific genes and their correlation patterns involved in the human age-dependent microglial and oligodendrocytic co-expression networks show only modest preservation in mice. Notably, only the human modules were significantly enriched for genetic variation associated with AD risk. This implies that microglial and oligodendrocytic ageing might contribute to AD risk, whereby genetic variants that alter the response of these cell types to ageing in the human hippocampus might modulate the onset of AD pathogenesis. Although the influence of microglia on AD risk is well known,^47^ the relationship between oligodendrocytes and myelination on AD risk is less well understood. However, it has been suggested previously that individuals with white matter abnormalities show a higher risk of dementia,^48^ and disruption of the expression and epigenetic marks of oligodendrocytic genes is seen in AD and ageing.^10,49-54^

We highlight new genes not previously reported to be associated with AD and show that they might have a role in age-related responses. Nonetheless, we cannot discount the possibility that these modules are associated with AD owing to the same genes being involved both in the response to amyloid-β (Aβ)-associated cellular damage during the AD process and in the cellular response to ageing. The hub genes for these modules and transcription factors whose targets are enriched within these modules are putative drivers and potential targets to influence these AD-related modules.

### Validation by subsequent GWAS studies

A recent GWAS representing the largest association study for dementia identified *ANKH*, *GRN*, *PLEKHA1*, *SNX1* and *UNC5CL* as new genes of interest,^40^ which validates our analysis here, because we also identify these genes as putative risk genes for AD, with a much smaller sample size than in the new AD GWAS. We identified that the inorganic pyrophosphate transport regulator, *ANKH*, and *PLEKHA1* were expressed by ARM/DAM cells. ANKH is a transmembrane protein involved in pyrophosphate and ATP efflux, which inhibits excessive mineralization and calcification^55^ and is linked to altered mineralization of tissues, including calcification of the vasculature leading to atherosclerosis. Limited studies have investigated the role of *ANKH in vivo*, but one study addressing vascular calcification in a rat model reported that *ANKH* activity is suppressed by TNF-α-activated nuclear factor-κB in immune cells, which promotes a pro-inflammatory signature, suggesting a role for this gene in regulating inflammation associated with disease.^56^ Furthermore, study of a consanguineous family with a novel *ANKH* mutation showed mental retardation in combination with the expected ankylosis and mineralization of soft tissues, and provided the first evidence of the pathological effect of *ANKH* in the CNS.^57^ Recent work has also shown strong association of *ANKH* genetic variation with cognitive change in people with normal ageing and probable AD using fluorodeoxyglucose-PET.^58^  *GRN* was confirmed to be a risk gene for dementia^40^ and, more specifically, frontotemporal degeneration.^59^  *GRN* was expressed by microglia transitioning to the ARM/DAM state in our analysis. *GRN* is of interest to the microglial community because loss-of-function mutations have been shown to cause excessive activation of microglia, showing generally opposing effects to TREM2 loss-of-function mutations.^60^  *SNX1* is expressed at the highest level by oligodendrocytes. SNX1 is a member of the sorting nexin family (SNX) that is involved in endosomal protein sorting, and its dysfunction has been linked to neurodegenerative diseases, including AD.^61^  *UNC5CL* was also part of the age-dependent oligodendrocyte network but is close to the *TREM2* locus and might therefore be linked to *TREM2* SNPs.^40^

### Variation associated with longevity is enriched in a single-cell homeostatic microglial network

Although there are clearly differences between human and mouse microglial responses to ageing, the separation of genes into microglial functions afforded by our co-expression analysis of single-cell mouse microglial transcriptomes, combined with our analysis of longevity-associated genes, highlighted that homeostatic functions of innate immune cells might be a significant contributor to longevity.

Our analysis also highlighted that microglial transcriptomic changes during ageing reflect those induced by demyelination. The myelin debris released during demyelination is lipid rich and compacted, and thus is difficult to digest by microglia. This might contribute to microglial dysfunction, brain ageing and neurodegeneration. Potentially, the loss of microglial homeostatic functions during ageing, possibly triggered in the hippocampus by microglial activation in response to age-related demyelination, might disrupt brain homeostasis, ultimately leading to less healthy ageing and an earlier death. This is a novel suggestion but is not totally surprising, because markers of neuronal and axonal damage are positively associated with mortality in elderly individuals^62^ and homeostatic functions of microglia maintain neuronal function and neurogenesis.^63-66^ Additionally, by conducting this analysis and grouping co-expressed genes into modules enriched for associations to specific biological annotations and populations of microglia, we provide a resource for researchers to gain a preliminary idea of a potential function of their gene of interest, simply by looking up in which module it is expressed. Given that this approach was no doubt hampered by the sparse nature of scRNA-seq data, we foresee it to become even more capable to distinguish groups of co-expressed genes involved in similar cellular processes as technology improves.

We particularly highlight a potential role for microglia in mediating at least some of the impact of *FOXO3* on longevity, which requires further exploration. *FOXO3* has previously been reported to augment microglial proliferation, activation and apoptotic injury.^67^ Therefore, lower *FOXO3* levels could lead to longevity, as indicated by our TWAS of whole-blood samples, by reducing loss of homeostatic myeloid cells during ageing. Additionally, we highlight the role of *CASP8* in homeostatic microglia. CASP8 is a protease with a well-known critical role in cell death mechanisms, such as inflammation, necroptosis and apoptosis.^68-70^  *CASP8* expression has been associated with several neurodegenerative disorders, in particular AD, Parkinson’s disease and autoimmune disorders.^68,69,71,72^ Several studies demonstrated that lipopolysaccharide-primed microglia promoted microglial proliferation, survival and pro-inflammation in a CASP8-dependent manner, associated with neurotoxicity towards neurons.^68,69,73^  *CASP8* has been reported to mediate Aβ-induced neuronal apoptosis, leading to the characteristic neuronal loss in AD.^71^ In a recent study, CASP8 was found to be active in multiple sclerosis lesions.^72^ Surprisingly, although microglial *Casp8* ablation in experimental autoimmune encephalomyelitis mice did not affect the course of disease, myeloid *Casp8* ablation worsened the autoimmune demyelination, because CASP8 acts as a negative regulator of the RIPK1/RIPK3/IL-1β pathway. Finally, a decline in T-cell numbers and impaired antibody production in B cells were observed in mice in the absence of *Casp8*,^74^ emphasizing its importance in immunity. These findings suggest a cell/tissue-specific role of *CASP8* in the body. Our TWAS data indicate that lower levels of *CASP8* in the cortex and cerebellum are associated with longevity, which might act by attenuating chronic neuroinflammation and cell death in the brain and thus promote longevity. However, we also saw in our TWAS data with whole blood the opposite effect to that in the cortex, whereby higher *CASP8* levels were associated with longevity, suggesting that *CASP8* might be beneficial for maintaining the innate and adaptive immune function of peripheral blood cells. It might be that the opposing effects seen for *CASP8* in cortex versus whole blood could be a direct effect of *CASP8* in promotion of longevity in one cell type, followed by a secondary, indirect compensatory response in the other cell type.

Collectively, our bulk and single-cell analyses suggest a new hypothesis, whereby genes associated with longevity in the human population might be most active in homeostatic microglial processes, whereas genes associated with AD are involved in responses to substantial disturbances of CNS homeostasis, such as age-related myelin breakdown and amyloid pathology (Fig. 7). The microglia-expressed genes might govern whether microglia can resolve myelin degradation and facilitate healthy ageing or whether microglia are overwhelmed by this challenge, leading to CNS dyshomeostasis and, potentially, then to AD. This two-hit hypothesis involving control of microglial homeostatic functions, then activation of microglia to support oligodendrocyte function might underlie how ageing and AD are linked. Discovering these genes showing variation associated with age might allow us to predict ‘physiological age’ versus ‘chronological age’ in the brain, and thus to predict AD and other age-related diseases better. These insights might also lead to intelligent design of new biomarkers to track disease progression and new mechanistic insights for drug development.

### Oligodendrocyte function in ageing and Ad

Interestingly, changes in the expression of microglial co-expression modules showed remarkable similarity between ageing and responses to demyelination in young mice. This indicates that microglial responses during ageing could be driven by age-related demyelination. Therefore, it might be that the microglial response to age-related demyelination disrupts their longevity-associated homeostatic networks. Likewise, human AD microglia, HAMs, seen in human AD patients have similar transcriptional profiles to microglial populations detected in multiple sclerosis patients, suggesting the possibility that demyelination might be a common phenomenon in ageing, multiple sclerosis and AD.^75^

Early changes in myelination and oligodendrocytes have been identified with AD,^52,53,76-78^ including induction of a gene co-expression network within oligodendrocytes in response to early amyloid pathology, with hub genes such as *Plp1*, *Mobp*, *Apod* and *Plekhb1*,^79^ thus showing a strong overlap with the ageing-dependent oligodendrocytic networks we identified. Recent work has also shown that *APOE* status affects myelination and oligodendrocyte function.^80,81^ Myelin debris is difficult to digest by microglia, and so might contribute to microglial dysfunction, ageing and neurodegeneration. In leukodystrophies (myelin degeneration), activated microglia collect in ‘nodules’, providing further evidence of the importance of microglia in responding to changes in myelin and axon integrity. White matter-associated microglia have distinct transcriptional profiles, depend on TREM2 signalling and upregulate ARM-associated genes involved in lipid metabolism and phagocytosis (such as *Apoe*, *Cst7*, *Bm2* and *Ctsb*).^8^ This is consistent with the TREM2 receptor binding to a series of ligands related to myelin debris uptake, including apolipoproteins and anionic lipids. In our age-dependent human transcriptome networks, *TREM2* was central in the microglial network, alongside downstream mediators *TYROBP* and *SYK*, and this age-dependent network was enriched with genetic variation associated with AD. Individuals with homozygous loss of function of *TREM2* display Nasu–Hakola disease, in which leukoencephalopathy is a prominent feature. Amongst our putative risk genes from the ageing-associated TWAS analysis are *EIF2B2*, *NPC1* and *TUFM*, which show leukodystrophy as a feature in people inheriting mutations of these genes (leukoencephalopathy with vanishing white matter, Niemann–Pick disease and combined oxidative phosphorylation deficiency 4, respectively). Thus, promotion of myelin integrity and microglial activity to support remyelination is an emerging area of therapeutics for AD.^12,77,82^

### Limitations of the study

Our study has limitations. First, our results and conclusions are based upon bioinformatics analyses and require further validation by new experiments. Second, longevity is a property of the whole organism, whereas AD is mostly relevant to brain health/disease; however, given that AD genetic architecture is age dependent,^83^ it is likely that age-related changes in the body influence brain health and vice versa, as mentioned above. Third, we have restricted the gene-level analysis of GWAS data to gene boundaries that can miss some non-coding variation associated with genes of interest, thus making our findings conservative. Fourth, our gene-based analysis identifies the significance for each gene, without accounting for LD between SNPs in other genes. Therefore, when the LD is strong in a particular region (*APOE* and *MHC*), we identify a number of closely positioned genes as significant. Fifth, given the lack of enrichment of human AD risk genes in the mouse age-dependent microglial module, our analysis of mouse scRNA-seq data potentially missed some age-dependent AD-associated gene modules that might be detected in human data. However, as stated above, to our knowledge, ideal scRNA-seq data from human hippocampal microglia do not yet exist with well-powered, non-diseased individuals across the human lifespan. Integration of human scRNA-seq data from different individuals with often unbalanced covariates of participants from which microglia can be extracted, such as pathology, can make the identification of age-related modules difficult. This can be seen by the incomplete overlap of age-related differentially expressed genes reported by bulk RNA studies of human microglia.^42,84-87^ Sixth, the use of monocyte data as a proxy for microglial gene expression might be imprecise because there are some transcriptional and phenotypic differences between monocyte-derived brain macrophages and microglia.^88^ It is also possible that the gene drivers we identify impact peripheral immune cell function to contribute to ageing and AD. Seventh, the current pace of GWAS is fast, and new GWAS will be available soon. Here we used the AD GWAS of Kunkle *et al*.^17^ and compared our putative risk genes with the newer AD GWAS of Bellenguez *et al*.^40^ Eighth, most GWAS datasets are from Caucasian individuals, meaning that, particularly for human longevity, which is highly affected by population differences, the gene variants identified might be dependent on ethnicity. Finally, given that the gene network module size and connectivity are influenced by parameter selection, with different sets of parameters the specificity of the modules could be altered, in contrast to the standard parameters we used here.

## Conclusion

In conclusion, we identify co-expression networks in the ageing brain that are enriched for genetic variation associated with AD or longevity. We show that genetic risk for AD is enriched in age-dependent activated microglia and oligodendrocytes. In contrast, we show that genetic variation associated with longevity is enriched in homeostatic microglia. Thus, genetic variants associated with longevity and AD both fall on microglia but control different and opposing states of their function (homeostatic versus activated). Understanding the function of these priority risk and hub genes might allow us to develop interventions to slow age-related brain decline and AD. These genetic networks that drive ageing, AD and inflammageing might help to predict the physiological age of the brain and AD progression more accurately in individual people by generation of biomarker and genotyping assays.

## Supplementary Material

awae339_Supplementary_Data

## Data Availability

The R code for performing the data analysis described in this manuscript is freely available via GitHub: https://github.com/AndyCGraham/AgeingCoExpNets. The data used for this study are publicly available for non-commercial research purposes; accession numbers and hyperlinks are given in the ‘Materials and methods’ section and the Supplementary material.
