# Supplementary material for: Human longevity and Alzheimer’s disease variants act via microglia and oligodendrocyte gene networks
Source: Brain. 2025 Jan 9;148(3):969–84. doi: 10.1093/brain/awae339 (PMC11884759; doi:10.1093/brain/awae339)
Supplement: awae339_Supplementary_Data [file awae339_supplementary_data.zip › brain-2024-00123-File010.pdf]

**Genetic variation associated with human longevity and Alzheimer's disease risk acts through core mammalian microglia and oligodendrocyte gene networks**

Andrew C. Graham[1†], Eftychia Bellou[2†], Janet C. Harwood[3], Umran Yaman[1], Meral Celikag[1], Naciye Magusali[1], Naiomi Rambarack[1], Juan A. Botia[4,5], Carlo Sala Frigerio[1], John Hardy[1,5], Valentina Escott-Price[3*], Dervis A. Salih[1*]

## Supplementary Materials

## Supplementary Methods.

### Gene-based analysis.

The gene-based approach we used summarizes the strength of the association of multiple adjacent SNPs restricted to individual gene boundaries (sequence between the first and last exons, including the introns), and so accounts for common and complex DNA variants associated with a particular trait, *e.g.* if longevity is conferred by several (semi) independent SNPs within a locus, each with moderate effect sizes[1,2]. The SNP-wise mean model in MAGMA was used for the analysis. SNPs were assigned to genes based on the location obtained from the GENCODE[3] (build 37). All required files can be downloaded from the software's website (https://ctg.cncr.nl/software/magma).

### RNA-seq data pre-processing

Transcripts per million (TPM) normalised bulk RNA-seq datasets generated from wild-type C57BL/6J mice (8-, 16-, 32-, and 72-weeks-of-age; data available from Mouseac.org)[4], and non-diseased human hippocampi (from 196 individuals; v8 TPM

counts downloaded from gtexportal.org/home/datasets)[5], were filtered to remove genes with <5% variation between samples, or mean expression levels below 0.5 TPM in all experimental groups. As large differences in gene expression distributions between samples were detected in GTEx data, this data was quantile-quantile normalised using preprocessCore R package's *normalize.quantiles* function. These bulk RNA-seq datasets were then transformed by log2(normalised counts+1). Batch effects were analysed in bulk RNA-seq datasets by multidimensional scaling using the limma R package's *plotMDS* function to visualise sample clustering due to sequencing batch. Additionally, correlation of batch covariates to principle components of variation between samples, were identified using the swamp R package's *prince* function and the CoExpNets R package's *princePlot* function[6].

Bulk RNA-seq datasets generated from microglia isolated from the hippocampus of aged and adult wild-type C57BL/6J mice[7] (data available from publication), or the corpus callosum of C57BL/6J mice treated for zero, 5-, or 12-weeks with a demyelinating cuprizone diet[8] (data downloaded from GSO: GSE130627), were normalised using DeSeq2's estimatesizefactors function[9]. Genes with an average detection level <1.5 normalised counts were removed.

Normalised bulk RNA-seq datasets generated from the hippocampi of wild-type mice of different ages[10–12], used to assess module preservation, were downloaded from Synapse accession syn20808171, GSO: GSE110741, and GSO: GSE61918, respectively. Genes with an average detection level <0.5 normalised counts or between sample variation <5%, and samples detected as outliers by having p<0.05 result from the outliers R package's *grubbs.test* function were removed.

Raw counts generated by plate-based scRNA-seq of microglia isolated from the hippocampi of 3-, 6-, 12-, and 21-month-old wild-type mice (downloaded from GSE127893; non-hippocampal and $APP^{NL\text{-}G\text{-}F}$ samples were removed)[13], were filtered to remove cells which appeared either unhealthy (>5% of reads aligned to mitochondrial genes or <1500 transcripts or <1000 genes sequenced) or potential doublets (>3500 genes sequenced). Counts were then transformed by log2(normalised counts+1), and genes not detected in >98.5% of cells in all predefined clusters, or with between sample variation <4%, were removed. Batch effects were assessed by running downstream analysis and determining correlation of generated modules to sequencing plate.

As all datasets demonstrated batch effects, these were removed using limma's *removeBatchEffect* function. The effects of trait(s)-of-interest were preserved using this function's design argument. This function's covariate argument was also used to remove the effects of confounding covariates, extraction batch, sequencing batch, post-mortem interval, sex, cause of death, and centre of origin, from the GTEx dataset.

**Co-expression network analysis**

Module Construction

Co-expression analysis was performed on pre-processed datasets using CoExpNets' *getDownstreamNetwork* function. CoExpNets is an optimisation of the popular WGCNA package[14], which uses an additional k-means clustering step to reassign genes to more appropriate modules, producing more biologically relevant and

reproducible modules[6]. Module eigengenes (ME), identified by CoExpNets were correlated (Pearson's product moment correlation) to numeric traits of interest using CoExpNets' *corWithNumTraits* function, or to categorical traits using the *corWithCatTraits* function.

Biological annotation

Modules formed by co-expression analysis were assessed for enrichment of cell-type specific genes using CoExpNets' *genAnnotationCellType* function. Module enrichment for biological annotations was assessed using the Gprofiler2 R package's *gost* function[15]. The genes assigned to the module (ordered by correlation with the module eigengene), were input as the query argument and all genes expressed above the expression threshold (TPM = 0.5 for bulk RNA-seq datasets, and detection in >2.5% of cells in any cell cluster for scRNA-seq datasets), were used as the custom background. Predicted annotations were excluded, and p-values were Bonferroni corrected. Module expression by age was assessed by two-tailed Student's *t*-test of the mean expression of the module's 100 most central genes (genes with highest correlation with the module eigengene) in the processed O'Neil *et al.* (2018) dataset[7], while module expression in cuprizone treatment groups was assessed by one-way ANOVA followed by the pairwise comparison of cuprizone diet timepoints to control diet using Dunnett's test, if the ANOVA indicated a significant difference between treatment groups ($p < 0.05$), in the processed Nugent *et al.* (2020) dataset[8].

Module preservation

Module preservation between our networks was calculated in pre-processed datasets described above, using CoExpNets' *preservationoneway* function. Control module

size was set to 400. This returned preservation statistics calculated by the WGCNA *modulePreservation* function[16], of which z.summary (summary of other preservation statistics), was reported unless the two sub-measures of preservation (z.connectivity and z.density) differed significantly. Comparison between human and mouse datasets was preceded by conversion of gene symbols to orthologues using the biomaRt R package[17]. Module eigengenes were detected in query datasets using CoExpNets *getNetworkEigengenes* function.

**Enrichment analyses of identified overlapping genes**

Enrichment analyses were performed by comparing the number of overlapping statistically significant genes (gene-based $p \leq 0.01$) showing association with AD or longevity derived from summary statistics from Kunkle *et al.* (2019)[18], and Timmers *et al.* (2020)[19], respectively, in our transcriptome module gene-sets with gene-sets randomly bootstrapped (Ninterations=100,000), from genes reliably expressed in the bulk RNA-seq (>0.5 TPM in any experimental group), and scRNA-seq datasets (expressed in >2.5% cells from any cluster). The random gene sets were matched to the gene sets of interest by: a) the number of genes in a set, and b) the numbers of independent SNPs per gene. The latter was estimated by MAGMA software as the number of principal components of the SNP data matrix of the gene, pruning away principal components with very small eigenvalues ensuring that only 0.1% of the variance in the SNP data matrix is pruned away. The enrichment analyses p-values (bootstrap p-values in the text) were calculated as the number of times when the random set of genes had larger or equal number of nominally significant genes in the module, divided by the total number of iterations. We report the bootstrap p-values based on genes' coordinates without 35 kb upstream and 10 kb downstream window

for two reasons a) to minimise the overlap between closely located genes due to their physical position, and b) to use the most conservative analysis as the gene-based p-values were more conservative without the 35 kb and 10 kb flanking windows. The bootstrap p-values do not require correction for multiple testing because the different age-associated gene modules were pre-selected from different transcriptome datasets (*i.e.* mouse versus human, bulk versus single-cell RNA-seq).

**Transcriptome-wise association study (TWAS)**

Expression weights were used in TWAS for autosomal chromosomes and excluding the MHC region, with the longevity summary statistics[19], using the R script FUSION.assoc_test.R from the FUSION software[20]. The TWAS weights were downloaded from the FUSION website (GTEX7 Brain (13 tissues), GTEX7 Whole blood)[5,21], YFS blood[22], NTR blood[23], and for all monocytes in the dataset produced by Fairfax *et al.* (2014)[24] (samples: CD14, LPS2, LPS24 and IFN-gamma), downloaded from https://github.com/janetcharwood/MONOCYTE_TWAS (see Harwood *et al.* (2021)[25] for details). Bonferroni correction for the number of genes analysed in each tissue was used to determine significance of TWAS associations. Plink v1.9 was used for SNP quality-control analysis, and MAGMA (v1.08) to obtain gene-wide p-values, and Logistic regression was performed in R.

## Supplementary Figures

**Supplementary Figure 1. Gene co-expression modules enriched for microglial and oligodendrocytic genes are significantly associated with age in the mouse hippocampus.** A) Co-expression analysis of bulk RNA-seq of the mouse hippocampus produced eight gene modules with significant correlation to age using our Mouseac data[4]. Pearson's product-moment correlation with age, value given in each cell where $R^2>0.4$, ** p <0.01, *** p < 0.001. No module has a significant association with either sequencing batch or lane, validating our batch correction strategy. B) Cell-type enrichment analysis reveals a significant enrichment of microglial genes in the microglial module, oligodendrocyte genes in the oligodendrocytic module, and dopaminergic neuron genes in the brown module. * p<0.05, ** p <0.01, *** p < 0.001. C) Network plot of the 151 most connected genes in the microglial module (left). Hub genes are shown in red. Biological annotations (right). D) Network plot of the 152 most connected genes in the oligodendrocytic module (left). Hub genes are shown in red. Biological annotations (right). Full networks given in Supplementary Table 3.

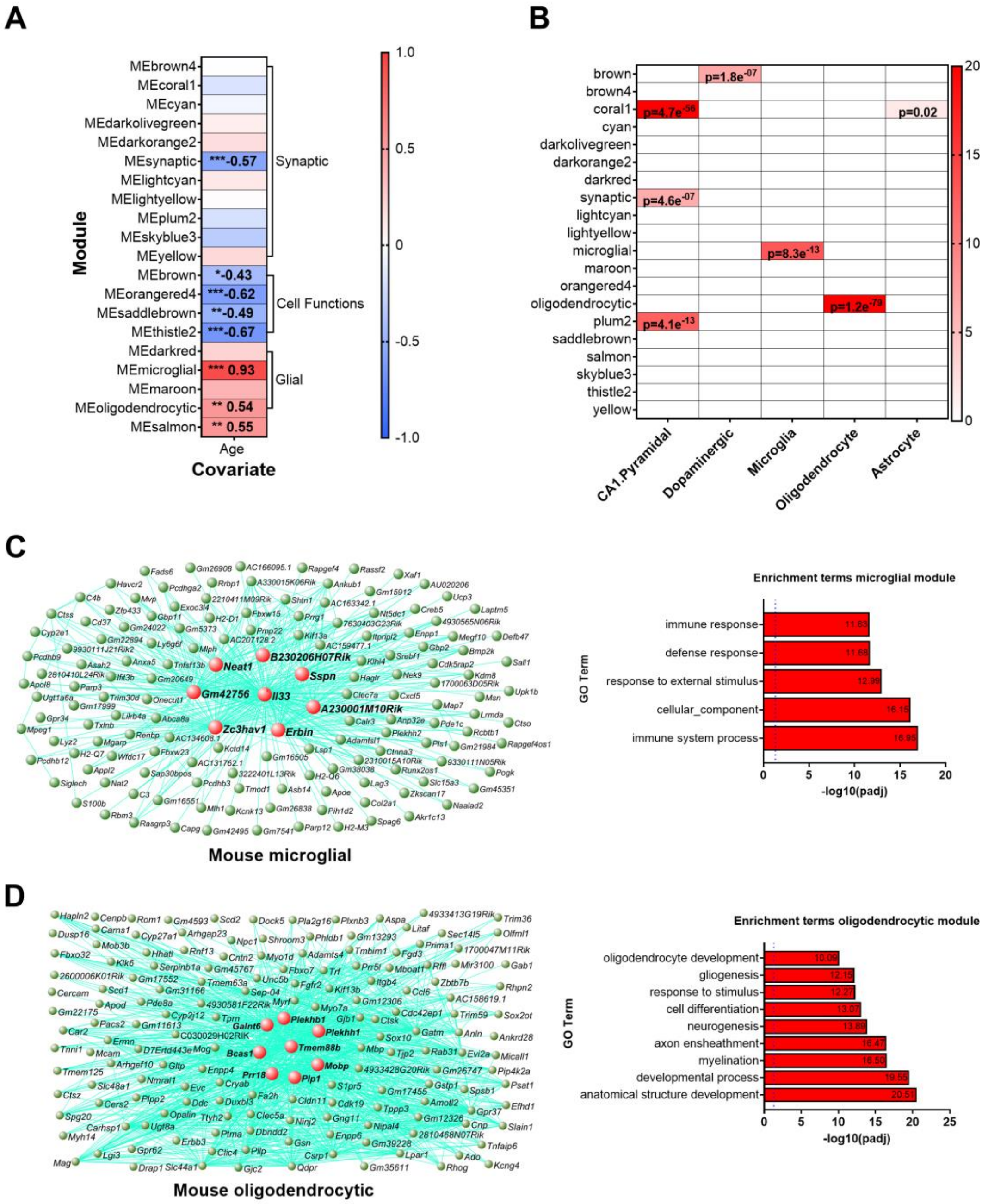


## Supplementary Figure 1

**Supplementary Figure 2. Genes within the mouse microglial module are expressed by oligodendrocytes.** Dot plot of the expression of the 14 most central genes within the mouse microglial bulk RNA-seq module (Supplementary Fig. 1c; 14 genes with highest correlation to the module eigengene) with respect to the different cell-types identified in scRNA-seq data of the wild-type mouse hippocampus at 3 and 24 months-of-age[26]. Supported by data from Ximerakis *et al.* (2019)[27].

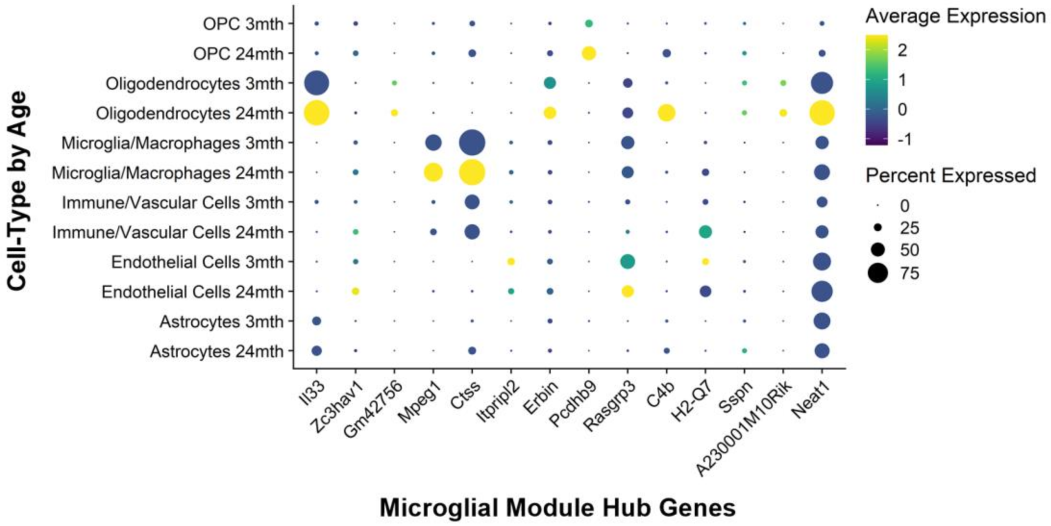


**Supplementary Figure 2**

**Supplementary Figure 3. Preservation of mouse microglial and oligodendrocytic bulk RNA-seq co-expression module's connectivity and correlation to age in publicly available datasets.** A) Preservation analysis determined that the mouse microglial and oligodendrocytic gene networks from our Mouseac data[4] both demonstrate strong preservation (z.summary > 10) of co-expression patterns between module genes in publicly available datasets generated by bulk RNA-seq of hippocampi from wild-type mice of different ages[10–12]. B) Module eigengene (ME) value (first principal component of module expression), calculated for our mouse microglial bulk module from our Mouseac dataset (black line; Salih *et al.* 2019)[4], alongside other datasets which include older mice, which all show strong positive correlations with age; Zhao *et al.* (2020)[10] (Pearson's product-moment correlation = 0.94, $p < 2.2x10^{-16}$), and Stilling *et al.* (2014)[12] (Pearson's product-moment correlation = 0.95, $p = 5.8x10^{-9}$) datasets. Individual data points represent the ME value in a single sample/mouse from that dataset. C) MEs calculated for our mouse oligodendrocytic module and other datasets also shows strong positive correlation to age in the datasets from Zhao *et al.* (2020)[10] (Pearson's product-moment correlation = 0.86, $p = 7.7x10^{-15}$), and Stilling *et al.* (2014)[12] (Pearson's product-moment correlation = 0.58, p = 0.01). Individual data points represent the ME value in a single sample/mouse from that dataset.

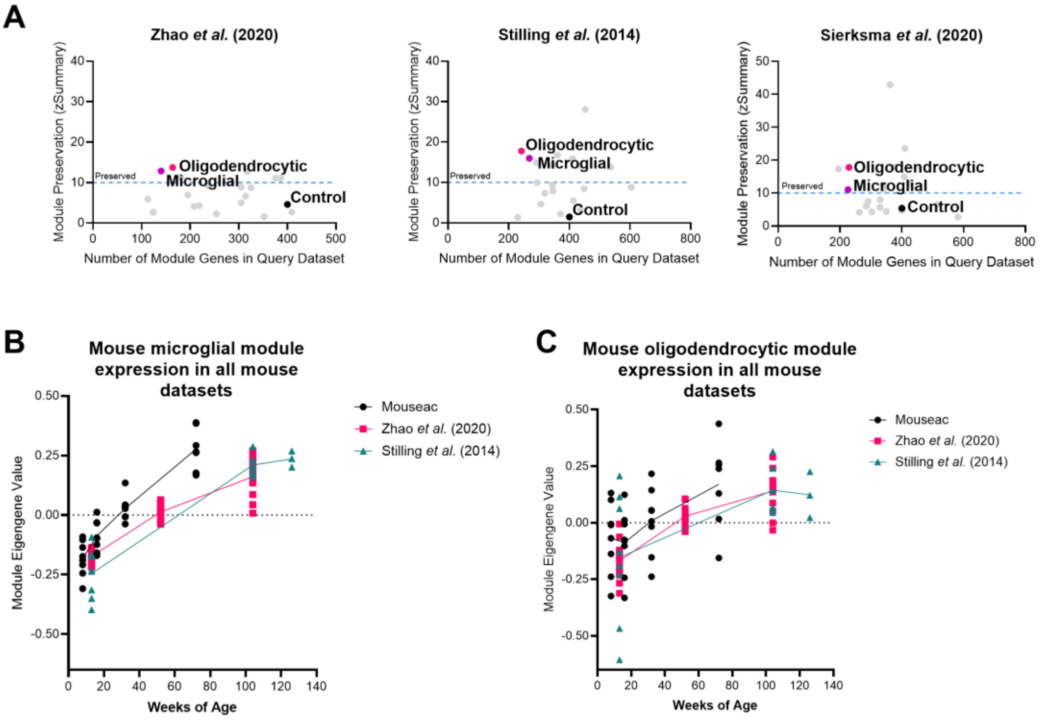


## Supplementary Figure 3

**Supplementary Figure 4. Enrichment of biological annotations associated with microglial age-dependent genetic module of genes expressed uniquely in mice are associated with antigen presentation, and genes expressed uniquely in humans are associated with leukocyte activation, cytokine production and T cell activation.** A) Enrichment terms associated with genes unique to the mouse microglial age-dependent module. B) Enrichment terms associated with genes unique to the human microglial age-dependent module.

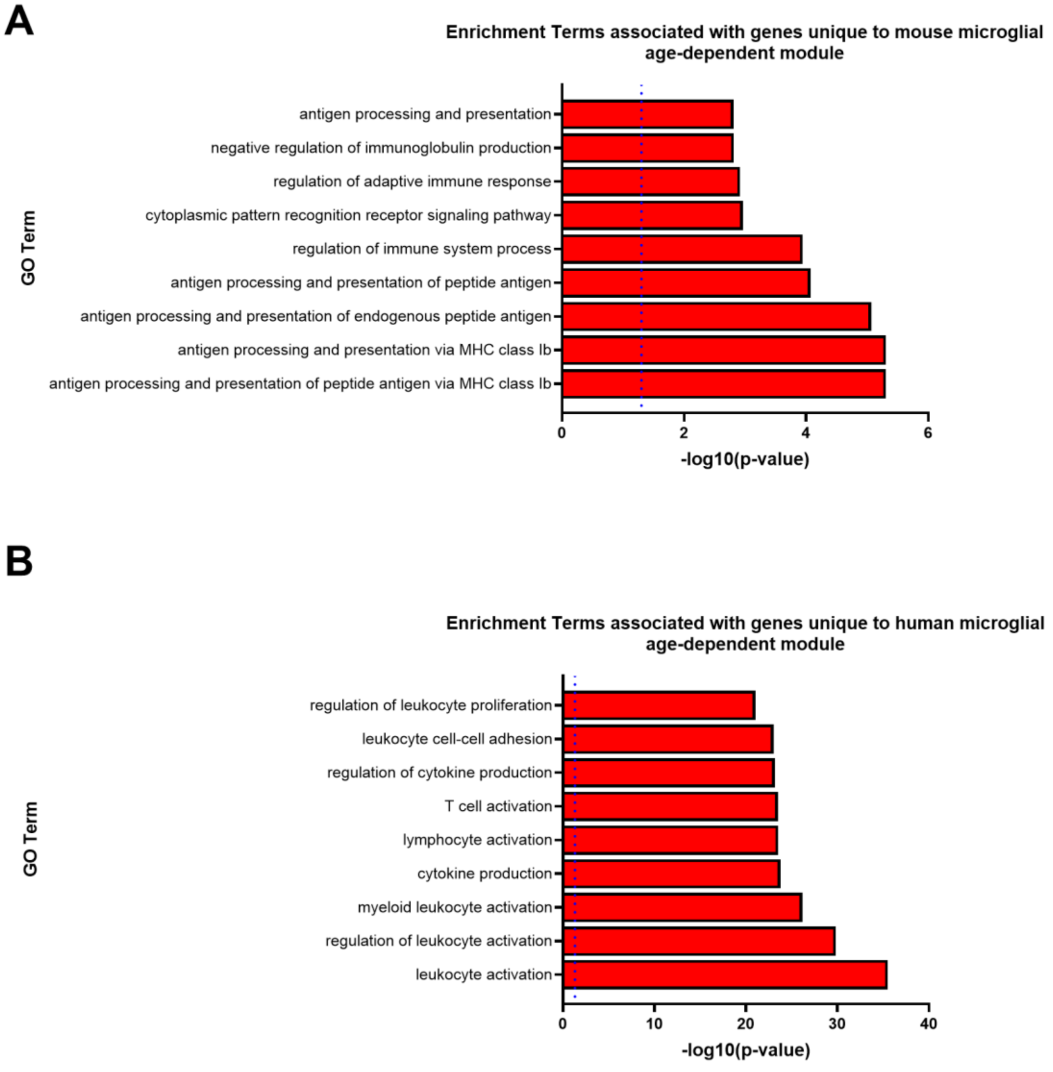


**Supplementary Figure 4**

**Supplementary Figure 5. Enrichment of biological annotations representing different aspects of the immune system within the mouse age-related scRNA-seq co-expression modules.** A) ARM-associated module. B) Interferon module. C) HM2-associated module. D) HM1-associated module. E) Phagolysosomal module. F) TGF-β module. G) Ribosomal module.

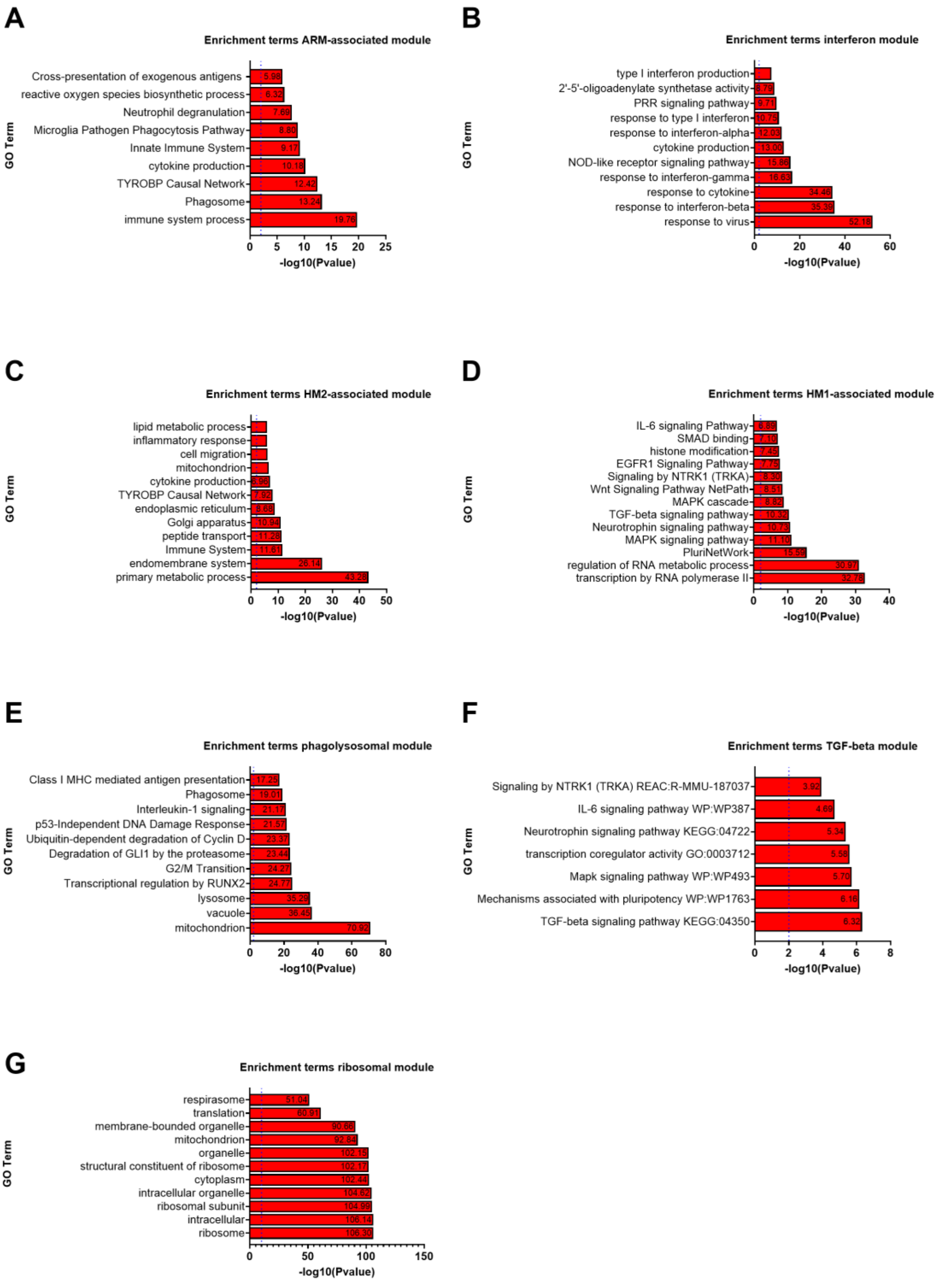


## Supplementary Figure 5

**Supplementary Figure 6. Gene co-expression modules expressed by distinct microglial subpopulations are dysregulated with age in the mouse hippocampus.** Expression of the differentially expressed genes in the Ribosomal (A), HM1-associated (C), and TGF-β (E) co-expression modules shown along this Pseudotime trajectory. B,D,F) Comparison of expression of the 100 most central genes (as a proxy for module expression) from the Ribosomal (B), HM1-associated (D), and TGF-β (F) modules in microglia isolated from the mouse hippocampus at 6-8 and 16-18 months-of-age and profiled by RNA-seq[7].

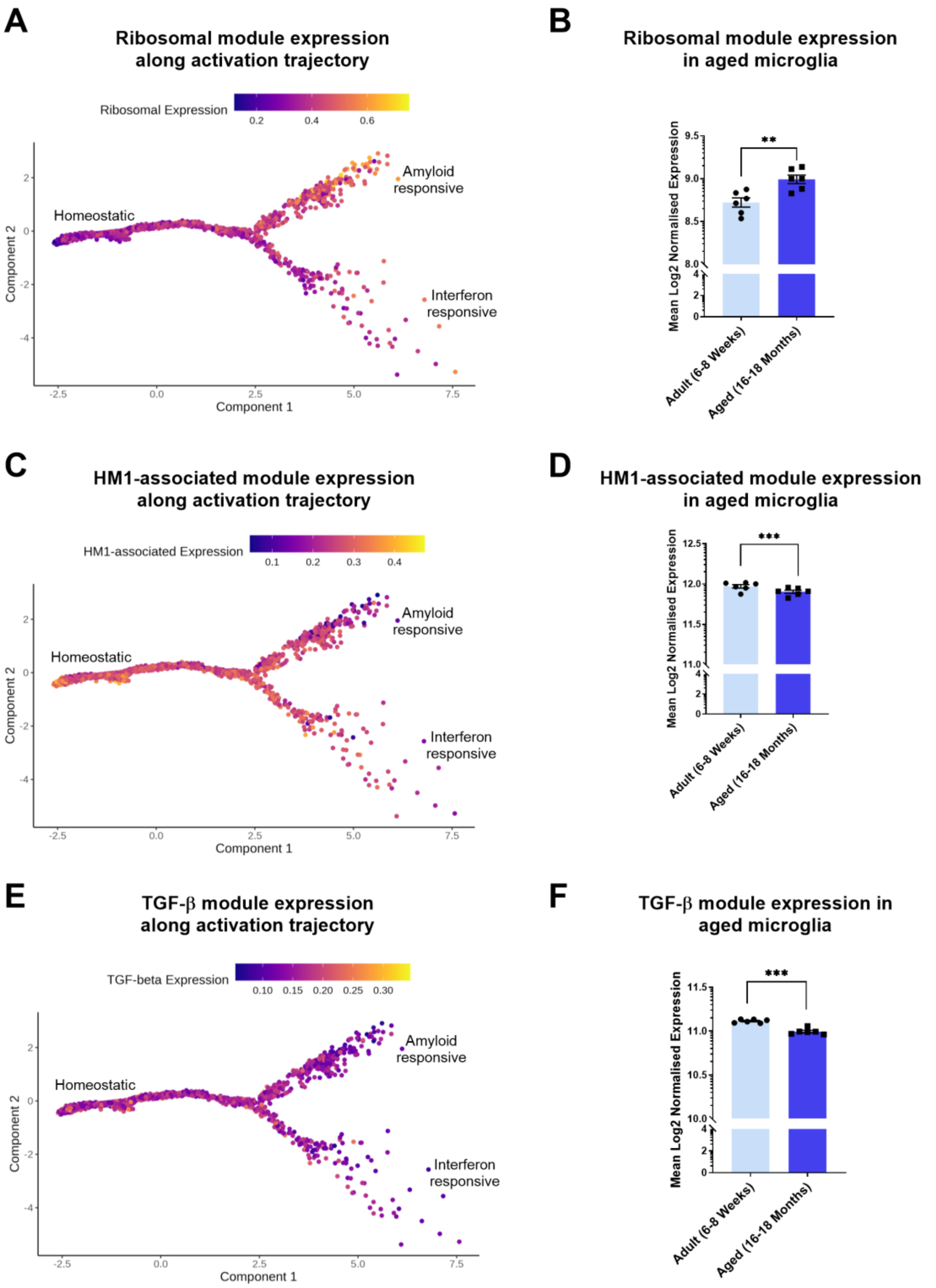


## Supplementary Figure 6

**Supplementary Figure 7. Manhattan plots of TWAS results identifying genes whose expression is associated with longevity in the GTEX7 brain tissues, GTEX7 whole blood[5,21], YFS blood[22], NTR blood[23], and for all monocytes in the dataset from Fairfax and colleagues (2014)[24] (samples: CD14, LPS2, LPS24 and IFN-gamma).** The y axis is the Z-score of the association between gene expression and longevity in the samples named in top left quarter of plot. Genes that showed significant association following Bonferroni correction for multiple testing are shown with red (ageing risk genes).

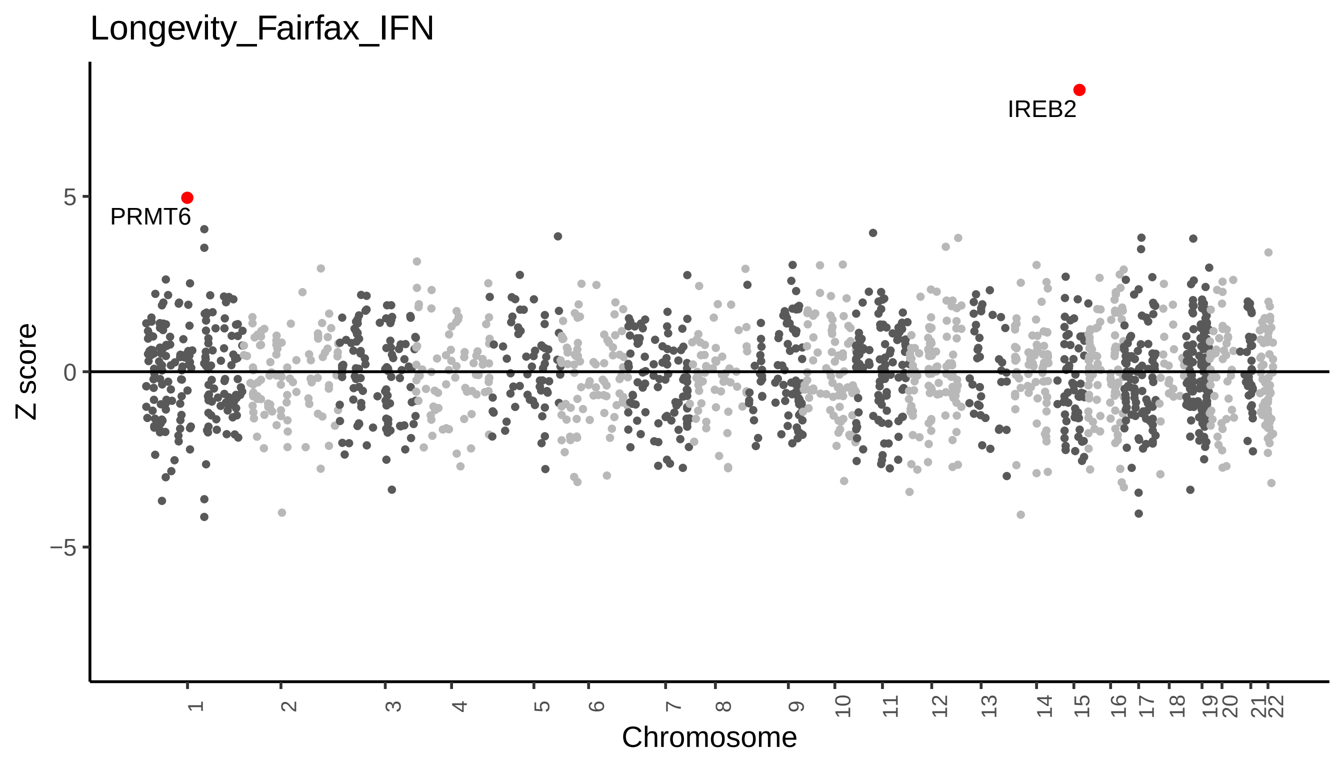

Longevity_Fairfax_IFN
IREB2
PRMT6
5
0
−5
Z score
1
2
3
4
5
6
7
8
9
10
11
12
13
14
15
16
17
18
19
20
21
22
Chromosome

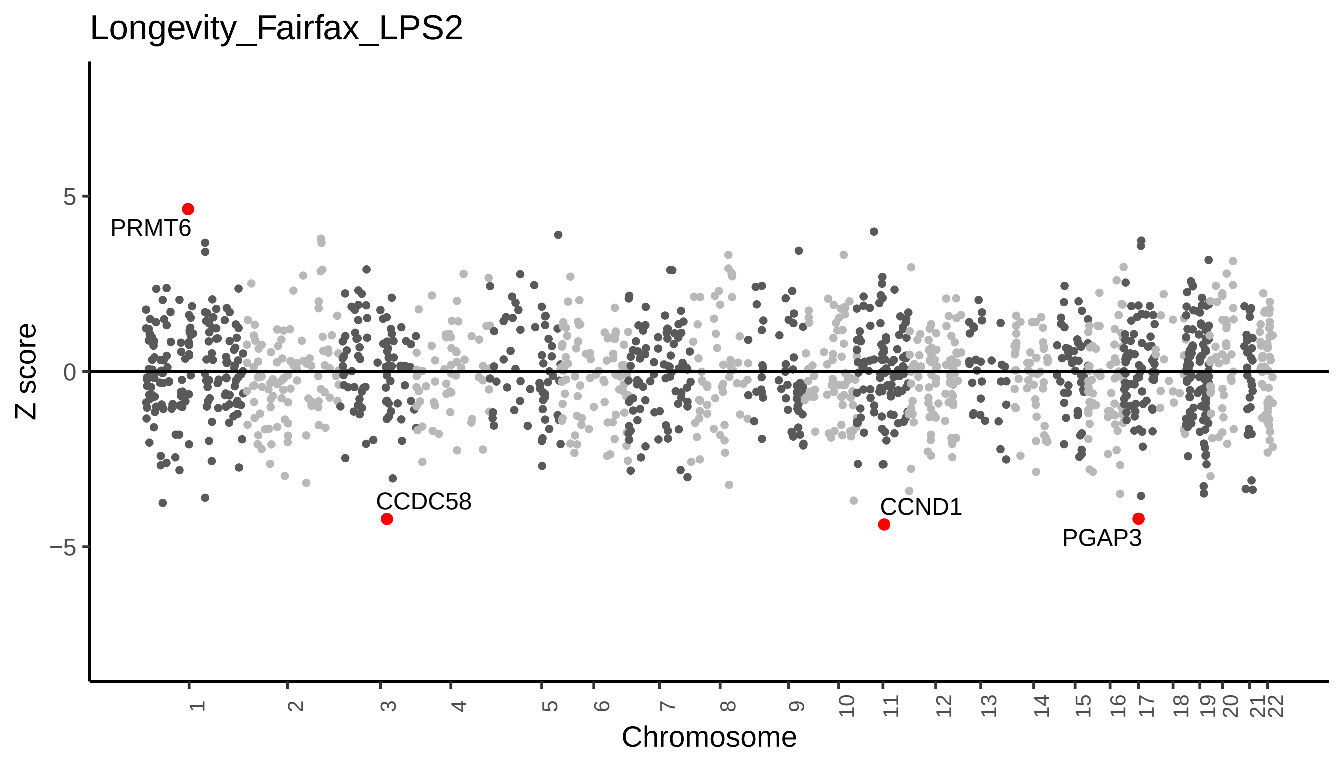

Longevity_Fairfax_LPS2
Z score
5
0
−5
PRMT6
CCDC58
CCND1
PGAP3
1
2
3
4
5
6
7
8
9
10
11
12
13
14
15
16
17
18
19
20
21
22
Chromosome

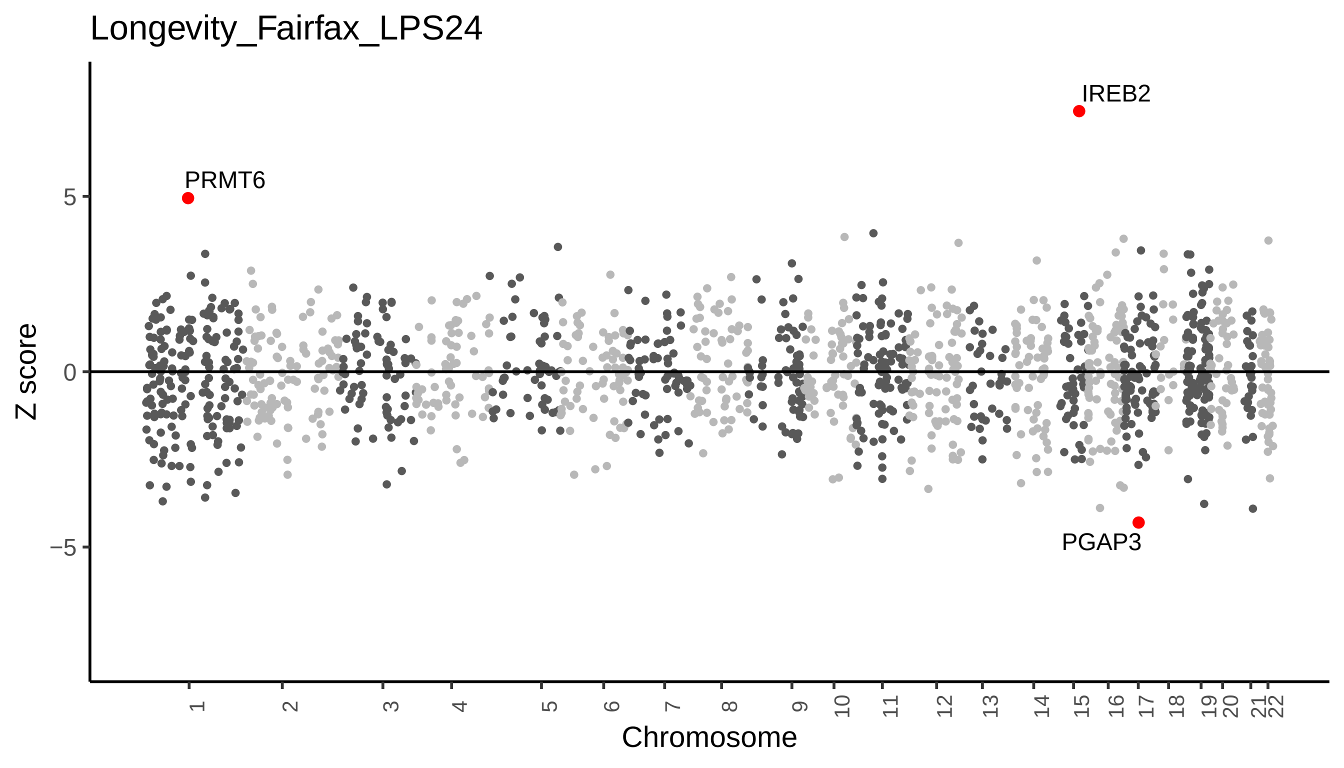
Longevity_Fairfax_LPS24
Z score
5
0
−5
PRMT6
IREB2
PGAP3
1
2
3
4
5
6
7
8
9
10
11
12
13
14
15
16
17
18
19
20
21
22
Chromosome

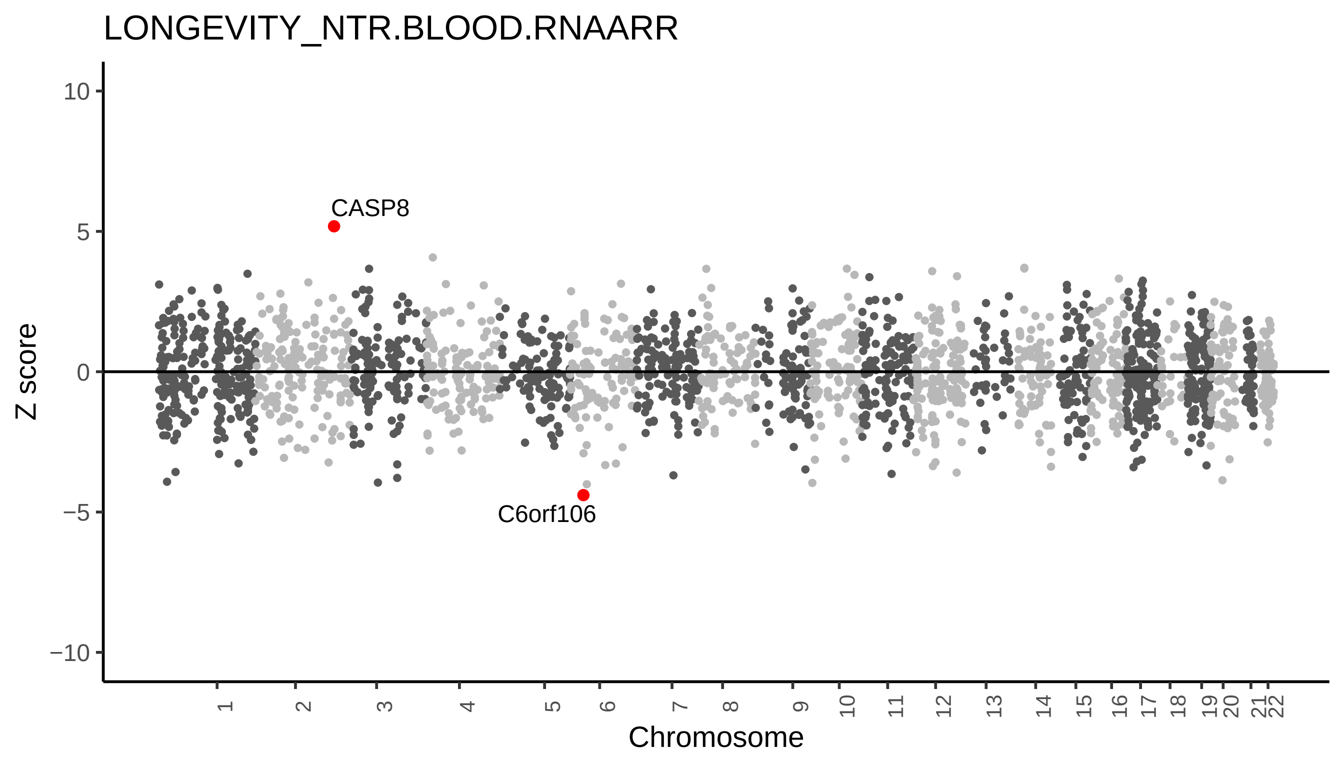
LONGEVITY_NTR.BLOOD.RNAARR
Z score
10
5
0
−5
−10
CASP8
C6orf106
Chromosome
1
2
3
4
5
6
7
8
9
10
11
12
13
14
15
16
17
18
19
20
21
22

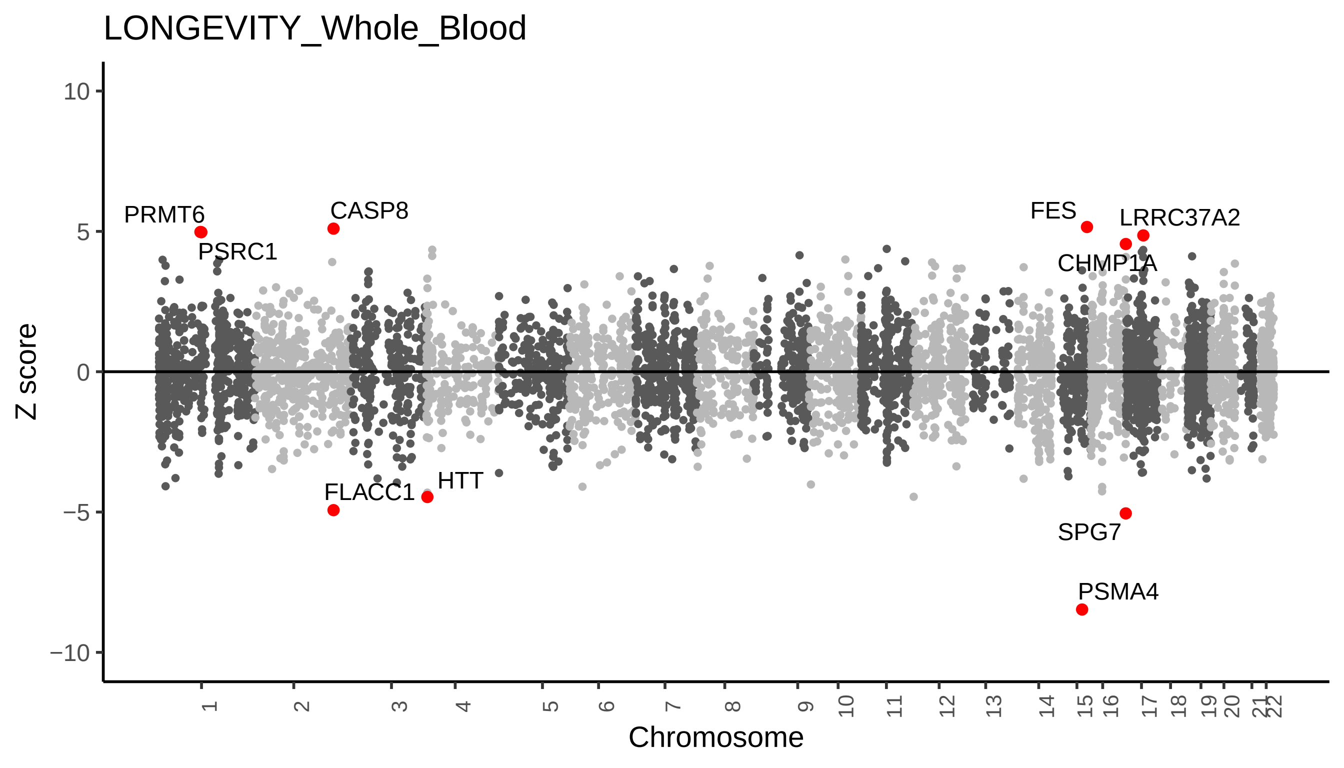

LONGEVITY_Whole_Blood
Z score
10
5
0
−5
−10
PRMT6
PSRC1
CASP8
FES
LRRC37A2
CHMP1A
FLACC1
HTT
SPG7
PSMA4
1
2
3
4
5
6
7
8
9
10
11
12
13
14
15
16
17
18
19
20
21
22
Chromosome

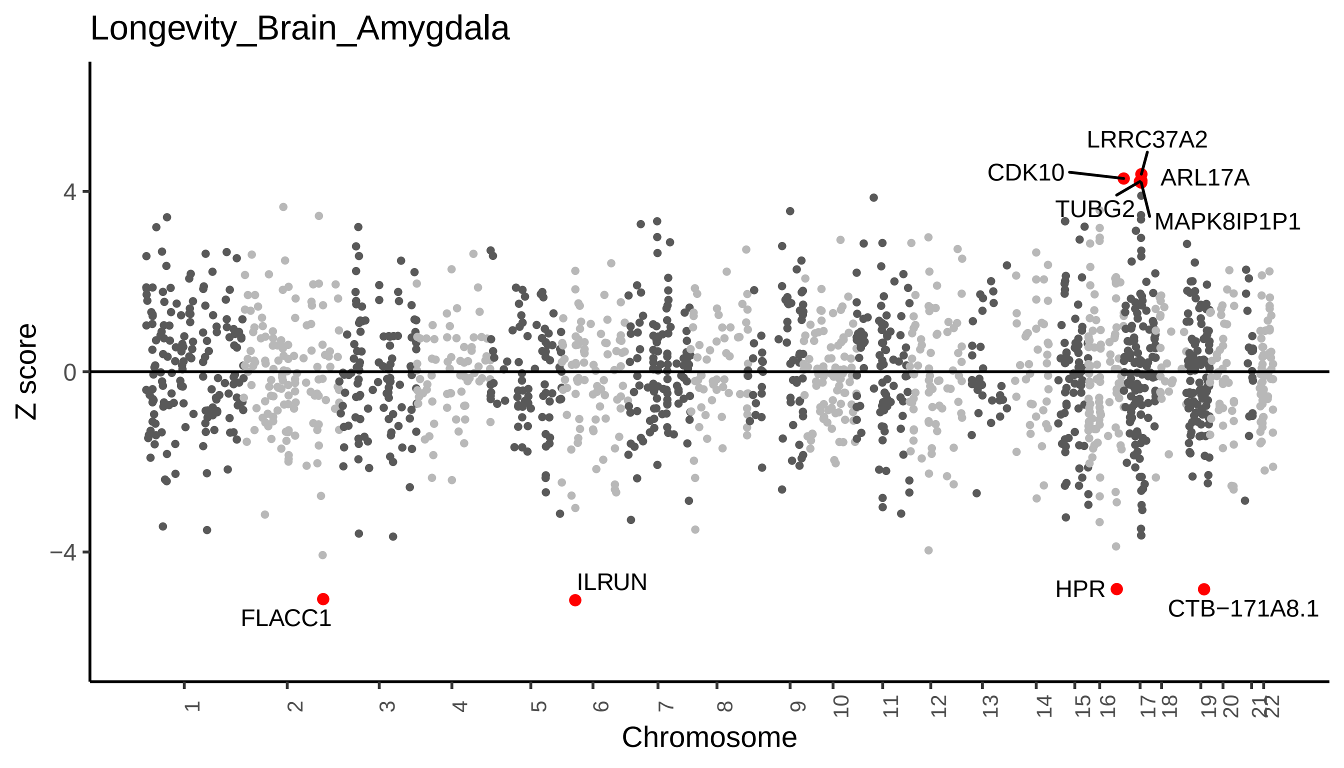

Longevity_Brain_Amygdala
Z score
4
0
−4
Chromosome
1
2
3
4
5
6
7
8
9
10
11
12
13
14
15
16
17
18
19
20
21
22
LRRC37A2
CDK10
ARL17A
TUBG2
MAPK8IP1P1
ILRUN
FLACC1
HPR
CTB−171A8.1

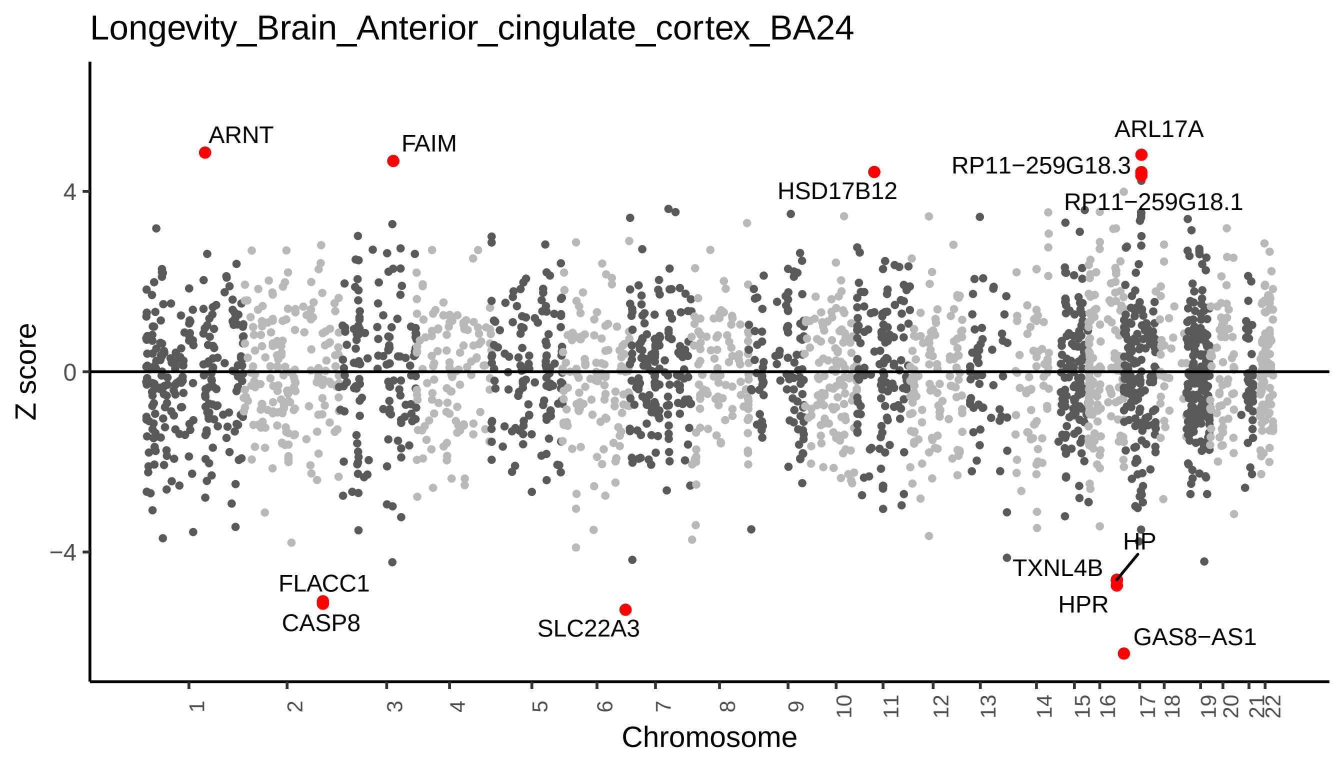
Longevity_Brain_Anterior_cingulate_cortex_BA24
Z score
4
0
−4
ARNT
FAIM
HSD17B12
ARL17A
RP11−259G18.3
RP11−259G18.1
FLACC1
CASP8
SLC22A3
HP
TXNL4B
HPR
GAS8−AS1
1
2
3
4
5
6
7
8
9
10
11
12
13
14
15
16
17
18
19
20
21
22
Chromosome

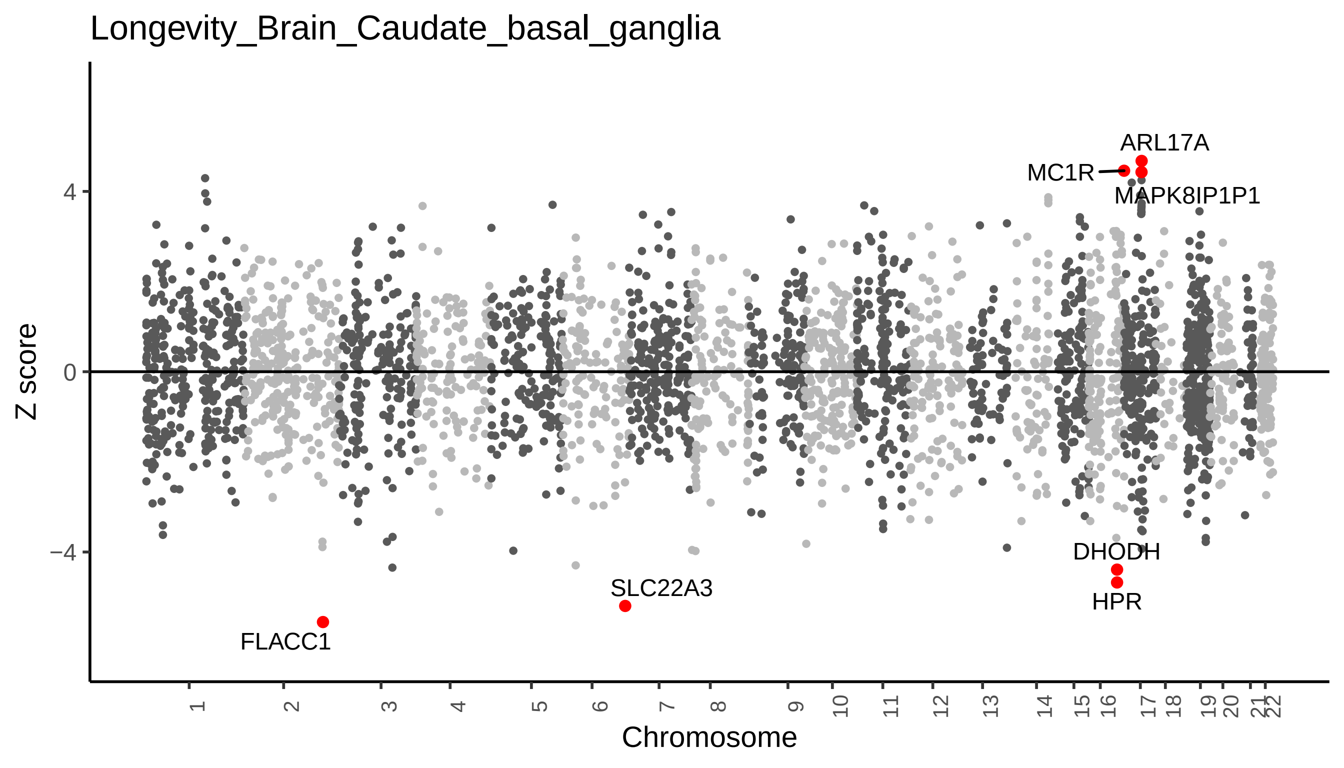

Longevity_Brain_Caudate_basal_ganglia
Z score
4
0
−4
Chromosome
1
2
3
4
5
6
7
8
9
10
11
12
13
14
15
16
17
18
19
20
21
22
ARL17A
MC1R
MAPK8IP1P1
DHODH
HPR
SLC22A3
FLACC1

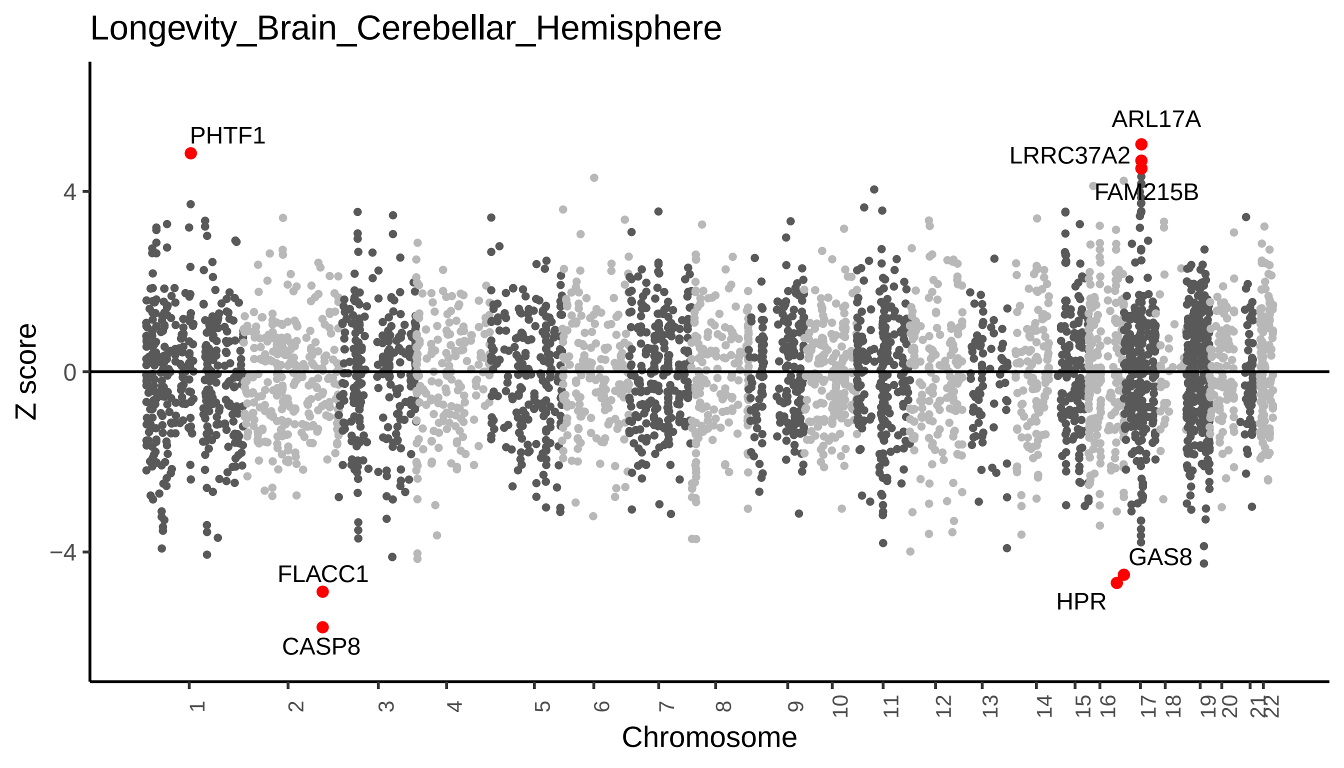

Longevity_Brain_Cerebellar_Hemisphere
PHTF1
ARL17A
LRRC37A2
FAM215B
FLACC1
CASP8
GAS8
HPR
4
0
−4
Z score
1
2
3
4
5
6
7
8
9
10
11
12
13
14
15
16
17
18
19
20
21
22
Chromosome

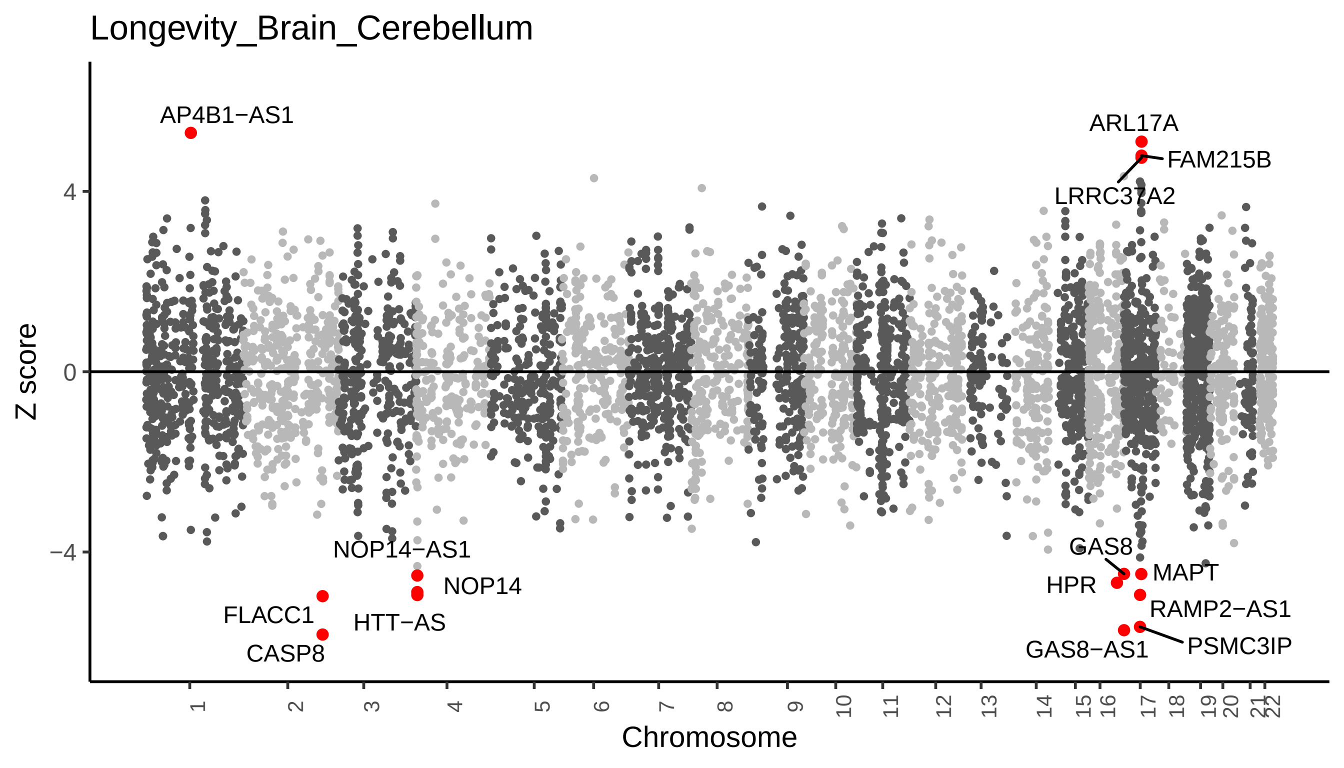

Longevity_Brain_Cerebellum
Z score
Chromosome
4
0
−4
1
2
3
4
5
6
7
8
9
10
11
12
13
14
15
16
17
18
19
20
21
22
AP4B1−AS1
ARL17A
FAM215B
LRRC37A2
NOP14−AS1
NOP14
HTT−AS
FLACC1
CASP8
GAS8
MAPT
HPR
RAMP2−AS1
PSMC3IP
GAS8−AS1

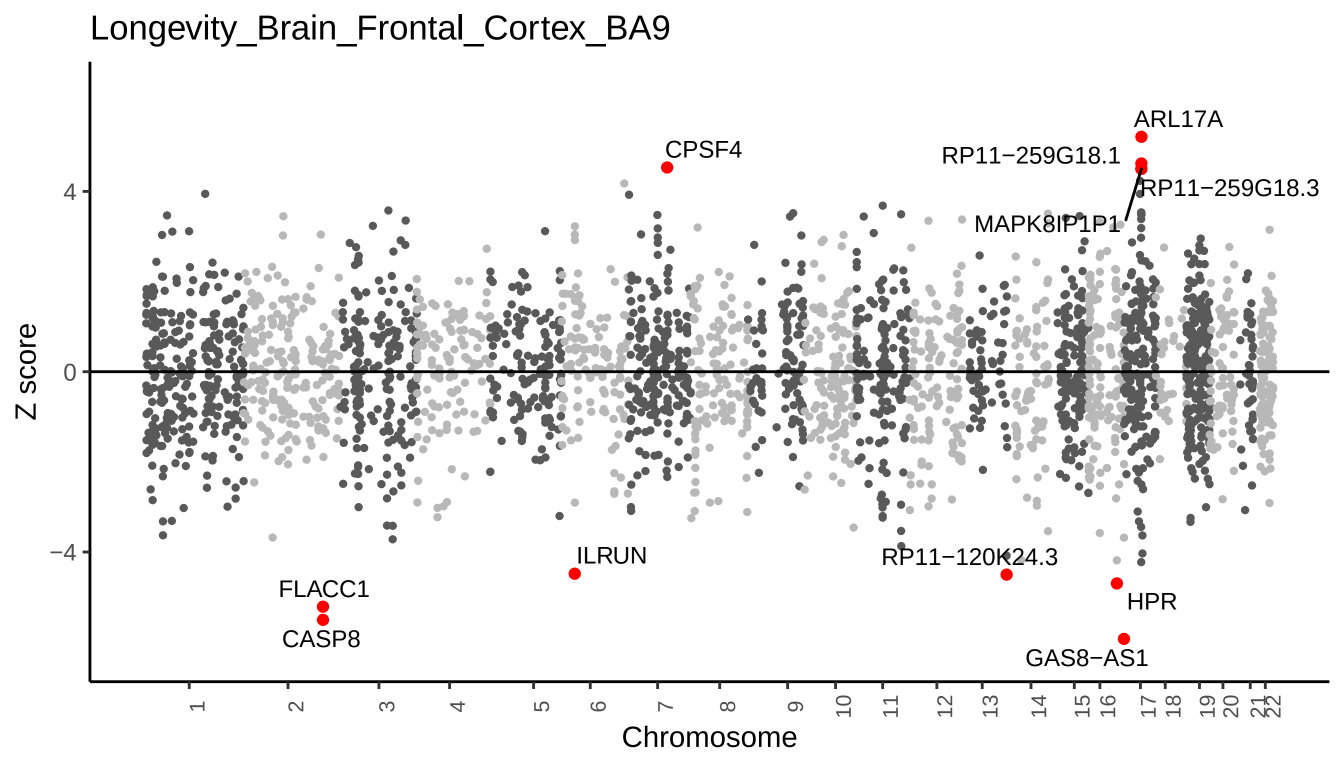

Longevity_Brain_Frontal_Cortex_BA9
Z score
4
0
−4
Chromosome
1
2
3
4
5
6
7
8
9
10
11
12
13
14
15
16
17
18
19
20
21
22
ARL17A
RP11−259G18.1
RP11−259G18.3
MAPK8IP1P1
CPSF4
ILRUN
RP11−120K24.3
HPR
GAS8−AS1
FLACC1
CASP8

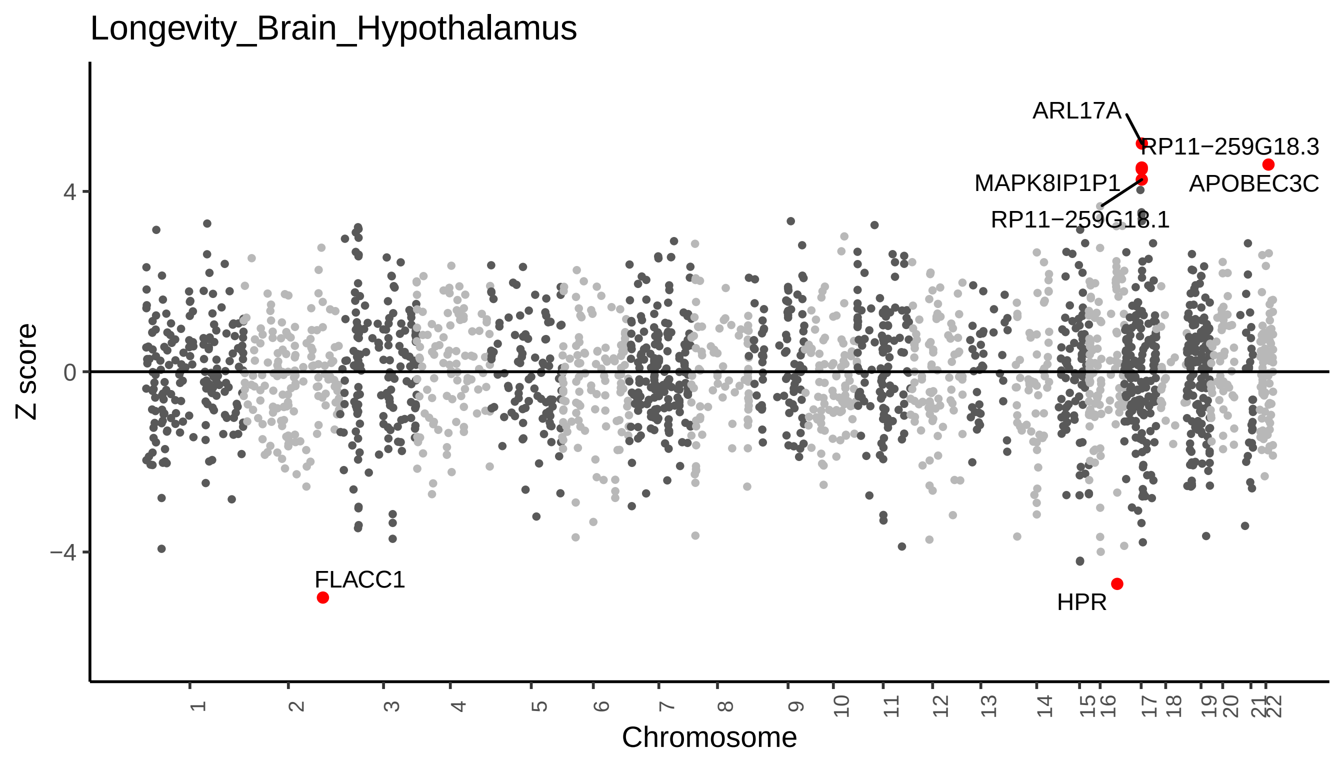

Longevity_Brain_Hypothalamus
Z score
4
0
−4
Chromosome
1
2
3
4
5
6
7
8
9
10
11
12
13
14
15
16
17
18
19
20
21
22
ARL17A
RP11−259G18.3
MAPK8IP1P1
APOBEC3C
RP11−259G18.1
FLACC1
HPR

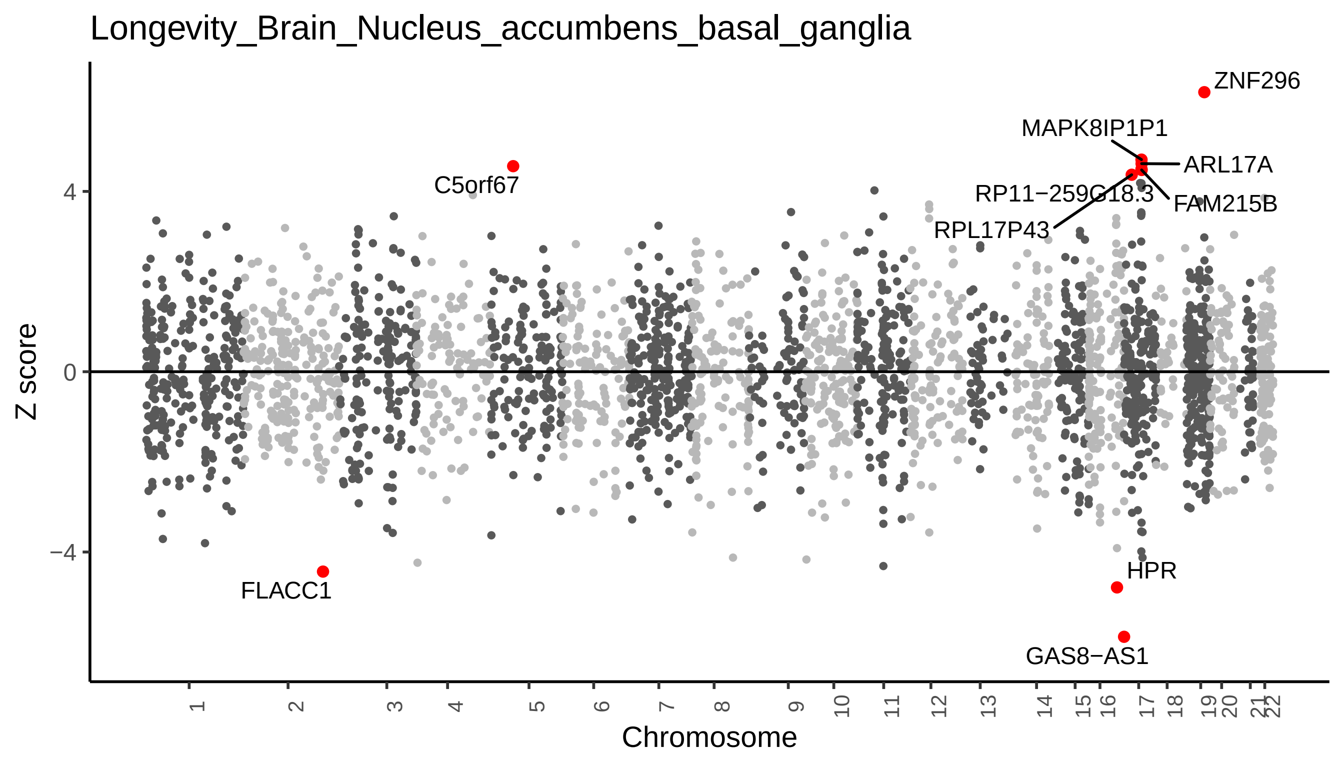

Longevity_Brain_Nucleus_accumbens_basal_ganglia
Z score
4
0
−4
Chromosome
1
2
3
4
5
6
7
8
9
10
11
12
13
14
15
16
17
18
19
20
21
22
ZNF296
MAPK8IP1P1
ARL17A
C5orf67
RP11−259G18.3
FAM215B
RPL17P43
FLACC1
HPR
GAS8−AS1

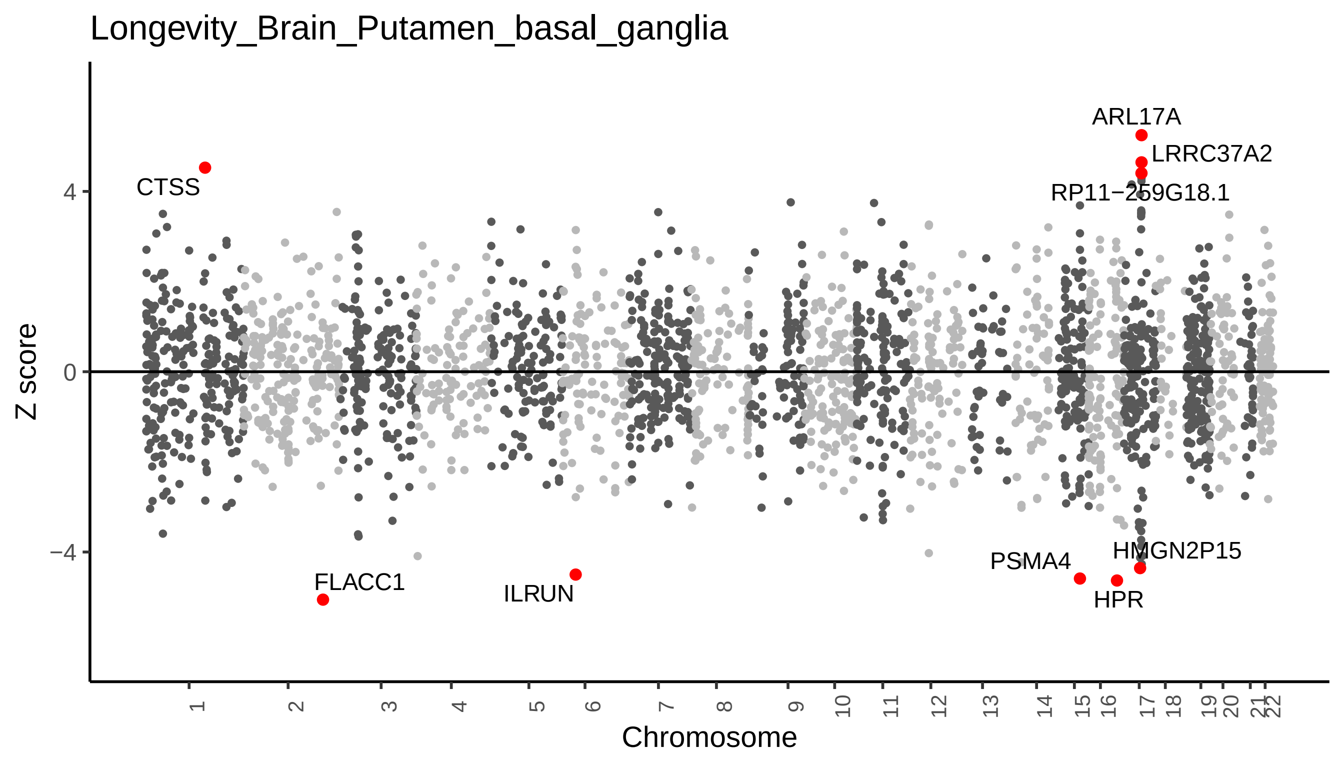

Longevity_Brain_Putamen_basal_ganglia
ARL17A
LRRC37A2
CTSS
RP11−259G18.1
4
0
−4
Z score
FLACC1
ILRUN
PSMA4
HMGN2P15
HPR
1
2
3
4
5
6
7
8
9
10
11
12
13
14
15
16
17
18
19
20
21
22
Chromosome

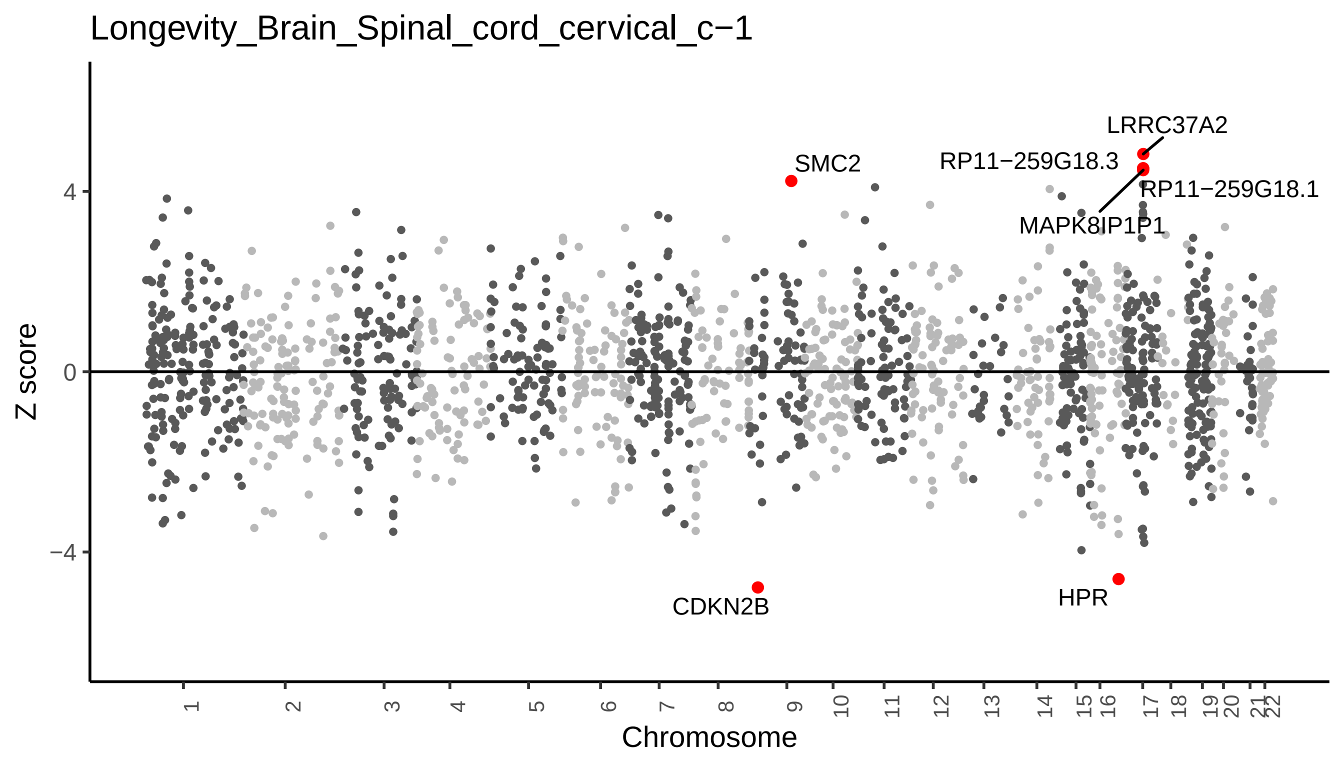

Longevity_Brain_Spinal_cord_cervical_c−1
Z score
Chromosome
4
0
−4
1
2
3
4
5
6
7
8
9
10
11
12
13
14
15
16
17
18
19
20
21
22
LRRC37A2
SMC2
RP11−259G18.3
RP11−259G18.1
MAPK8IP1P1
CDKN2B
HPR

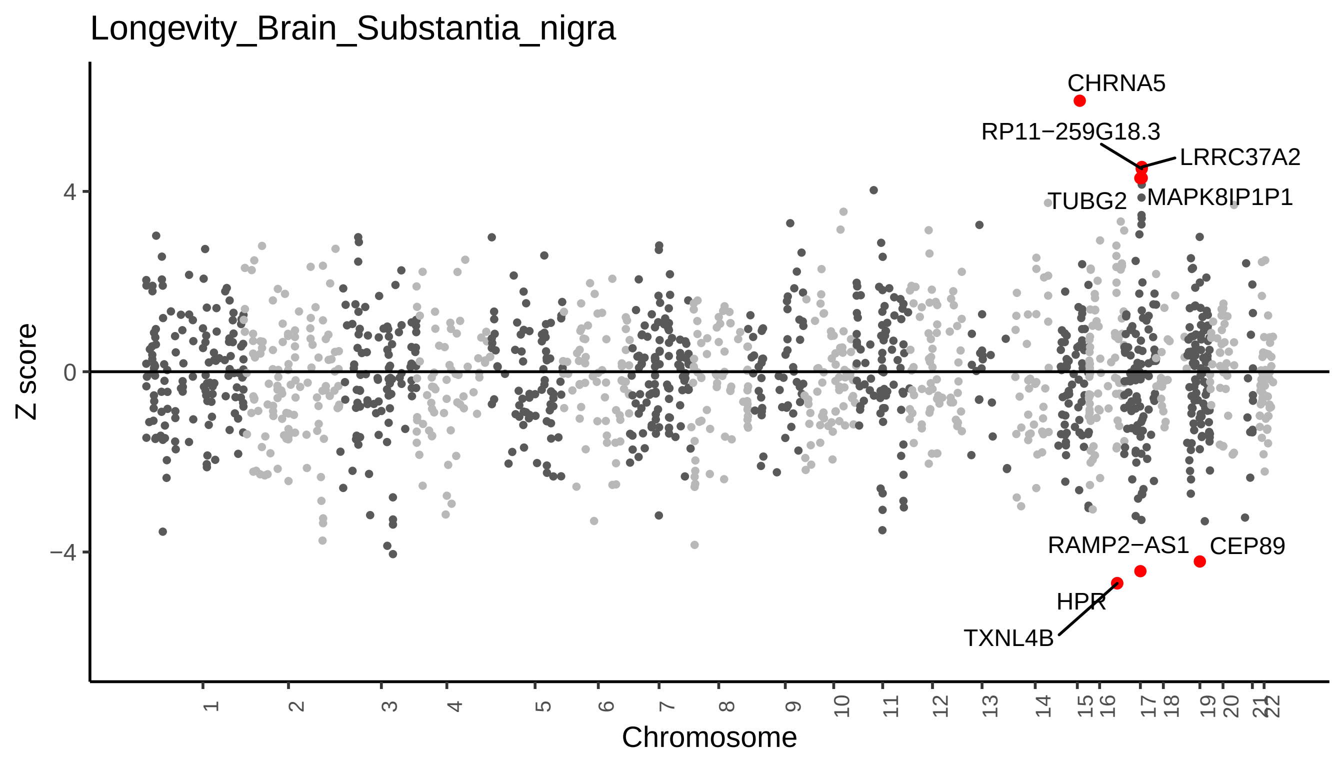
Longevity_Brain_Substantia_nigra
Z score
Chromosome
4
0
−4
1
2
3
4
5
6
7
8
9
10
11
12
13
14
15
16
17
18
19
20
21
22
CHRNA5
RP11−259G18.3
LRRC37A2
TUBG2
MAPK8IP1P1
RAMP2−AS1
CEP89
HPR
TXNL4B

**Supplementary Figure 8. Ingenuity plot showing the TWAS hits associated with longevity are linked in a network containing *APOE*.** Solid lines indicate direct interactions (*e.g.* protein-protein interactions, or phosphorylation), and broken lines indirect interactions, between pairs of genes across all mammalian species for tissues and cell types curated in Ingenuity (QIAGEN). Longevity-associated genes from TWAS used as input (P ≤ 0.01 and Z > 2.5 or < -2.5; Supplementary Table 8). Default settings used.

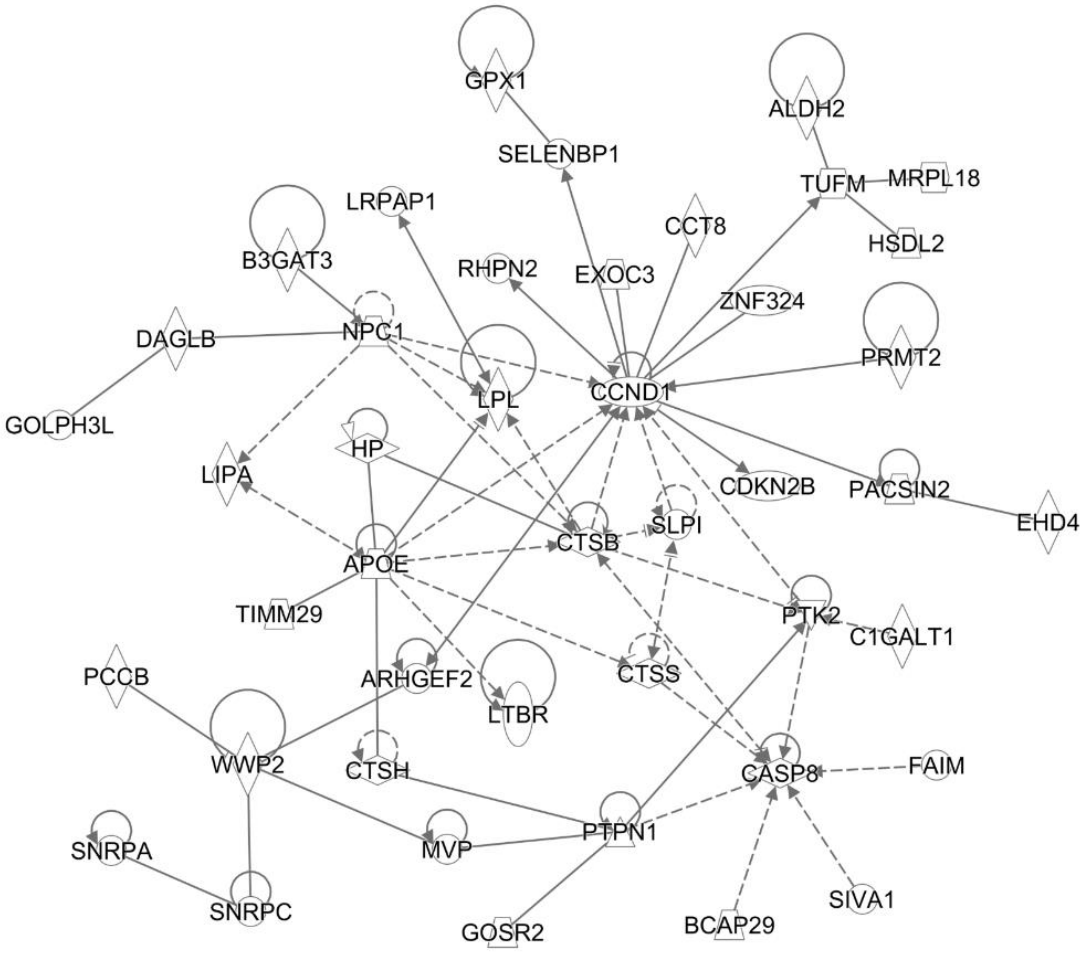

## References


1 de Leeuw CA, Mooij JM, Heskes T, Posthuma D. MAGMA: generalized gene-set analysis of GWAS data. *PLoS Comput Biol*. 2015;11:e1004219.

2 Escott-Price V, Bellenguez C, Wang L-S, et al. Gene-Wide Analysis Detects Two New Susceptibility Genes for Alzheimer's Disease. *PLoS One*. 2014;9:e94661.

3 Harrow J, Frankish A, Gonzalez JM, et al. GENCODE: the reference human genome annotation for The ENCODE Project. *Genome Res*. 2012;22:1760–74.

4 Salih DA, Bayram S, Guelfi S, et al. Genetic variability in response to amyloid beta deposition influences Alzheimer's disease risk. *Brain Commun*. 2019;1:fcz022.

5 Human genomics. The Genotype-Tissue Expression (GTEx) pilot analysis: multitissue  gene regulation in humans. *Science*. 2015;348:648–60.

6 Botía JA, Vandrovcova J, Forabosco P, et al. An additional k-means clustering step improves the biological features of WGCNA gene co-expression networks. *BMC Syst Biol*. 2017;11:47.

7 O'Neil SM, Witcher KG, McKim DB, Godbout JP. Forced turnover of aged microglia induces an intermediate phenotype but does not  rebalance CNS environmental cues driving priming to immune challenge. *Acta Neuropathol Commun*. 2018;6:129.

8 Nugent AA, Lin K, van Lengerich B, et al. TREM2 Regulates Microglial Cholesterol Metabolism upon Chronic Phagocytic Challenge. *Neuron*. 2020;105:837-854.e9.

9 Love MI, Huber W, Anders S. Moderated estimation of fold change and dispersion for RNA-seq data with DESeq2. *Genome Biol*. 2014;15:550.

10 Zhao N, Ren Y, Yamazaki Y, Qiao W, Li F, Felton LM. Alzheimer's Risk Factors Age, APOE, Genotype and Sex Drive Distinct Molecular Pathways. *Neuron*.

2020;106:727-742.e6.

11 Sierksma A, Lu A, Mancuso R, et al. Novel Alzheimer risk genes determine the microglia response to amyloid-β but not to TAU pathology. *EMBO Mol Med*. 2020;12

12 Stilling RM, Benito E, Gertig M, et al. De-regulation of gene expression and alternative splicing affects distinct cellular pathways in the aging hippocampus. *Front Cell Neurosci*. 2014;8:373.

13 Sala Frigerio C, Wolfs L, Fattorelli N, et al. The Major Risk Factors for Alzheimer's Disease: Age, Sex, and Genes Modulate the Microglia Response to Aβ Plaques. *Cell Rep*. 2019;27:1293-1306.e6.

14 Langfelder P, Horvath S. WGCNA: an R package for weighted correlation network analysis. *BMC Bioinformatics*. 2008;9:559.

15 Kolberg L, Raudvere U, Kuzmin I, Vilo J, Peterson H. gprofiler2 -- an R package for gene list functional enrichment analysis and namespace conversion toolset g:Profiler [version 2; peer review: 2 approved]. *F1000Research*. 2020;9:709.

16 Langfelder P, Luo R, Oldham MC, Horvath S. Is my network module preserved and reproducible? *PLoS Comput Biol*. 2011;7:e1001057.

17 Durinck S, Spellman PT, Birney E, Huber W. Mapping identifiers for the integration of genomic datasets with the R/Bioconductor package biomaRt. *Nat Protoc*. 2009;4:1184–91.

18 Kunkle BW, Grenier-Boley B, Sims R, et al. Genetic meta-analysis of diagnosed Alzheimer's disease identifies new risk loci and implicates Aβ, tau, immunity and lipid processing. *Nat Genet*. 2019;51:414–30.

19 Timmers PRHJ, Wilson JF, Joshi PK, Deelen J. Multivariate genomic scan implicates novel loci and haem metabolism in human ageing. *Nat Commun*. 2020;11:1–10.

20 Gusev A, Ko A, Shi H, et al. Integrative approaches for large-scale transcriptome-wide association studies. *Nat Genet*. 2016;48:245–52.

21 Consortium TGte, Aguet F, Anand S, et al. The GTEx Consortium atlas of genetic regulatory effects across human tissues. *Science (80- )*. 2020;369:1318–30.

22 Raitakari OT, Juonala M, Rönnemaa T, et al. Cohort profile: the cardiovascular risk in Young Finns Study. *Int J Epidemiol*. 2008;37:1220–6.

23 Willemsen G, de Geus EJC, Bartels M, et al. The Netherlands Twin Register biobank: a resource for genetic epidemiological studies. *Twin Res Hum Genet Off J Int Soc Twin Stud*. 2010;13:231–45.

24 Fairfax BP, Humburg P, Makino S, et al. Innate immune activity conditions the effect of regulatory variants upon monocyte gene expression. *Science (80- )*. 2014;343:1246949.

25 Harwood JC, Leonenko G, Sims R, Escott-Price V, Williams J, Holmans P. Defining functional variants associated with Alzheimer’s disease in the induced immune response. *Brain Commun*. 2021;3:fcab083.

26 Ogrodnik M, Evans SA, Fielder E, et al. Whole-body senescent cell clearance alleviates age-related brain inflammation and cognitive impairment in mice. *Aging Cell*. 2021;20:e13296.

27 Ximerakis M, Lipnick SL, Innes BT, et al. Single-cell transcriptomic profiling of the aging mouse brain. *Nat Neurosci*. 2019;22:1696–708.
