# Supplementary material for: Human longevity and Alzheimer’s disease variants act via microglia and oligodendrocyte gene networks
Source: Brain. 2025 Jan 9;148(3):969–84. doi: 10.1093/brain/awae339 (PMC11884759; doi:10.1093/brain/awae339)
Supplement: awae339_Supplementary_Data [file awae339_supplementary_data.zip › brain-2024-00123-File014.pdf]

Supplementary Table 4. Human hippocampus bulk RNA-seq microglial module genes and their gene-based analysis p-value for Alzheimer's disease.

Mouse Symbol	Human Symbol	Human Chromosome	Start Location	End Location	AD Gene P-value
Ms4a4a, Ms4a4b, Ms4a4c, Ms4a4d	*MS4A4A*	11	59953175	60085417	1.22E-14
Ms4a6c, Ms4a6b, Ms4a6d	*MS4A6A*	11	59939487	59952139	2.02E-12
Spi1	*SPI1*	11	47376411	47400127	8.98E-12
Apoc2, Gm44805	*APOC2*	19	45449239	45452822	9.62E-11
Trem2	*TREM2*	6	41126244	41130924	1.42E-08
H2-Eb2	*HLA-DRB1*	6	32546546	32557625	2.96E-07
-	*CD33*	19	51728320	51747115	1.05E-06
H2-Eb2	*HLA-DRB5*	6	32485120	32498064	1.19E-06
Inpp5d	*INPP5D*	2	233924677	234116549	8.14E-06
Arhgap45	*ARHGAP45*	19	1065922	1086627	2.40E-05
H2-Ab1	*HLA-DQB1*	6	32627244	32636160	2.92E-05
Laptm5	*LAPTM5*	1	31205316	31230667	6.64E-05
-	*HLA-DQA1*	6	32595956	32614839	3.96E-04
H2-Ea	*HLA-DRA*	6	32407619	32412823	9.98E-04
-	*NOP2*	12	6666029	6677857	1.32E-03
Atp8b4	*ATP8B4*	15	50150435	50475014	1.36E-03
Gpsm3	*GPSM3*	6	32158543	32163300	3.83E-03
Gal3st4	*GAL3ST4*	7	99756867	99766373	4.60E-03
Cmtm7	*CMTM7*	3	32433163	32524559	5.34E-03
Pced1b	*PCED1B*	12	47473386	47630445	5.61E-03
Itgam, Gm49368	*ITGAM*	16	31271288	31344213	5.77E-03
Dok3	*DOK3*	5	176928908	176938275	6.00E-03
Tmc8	*TMC8*	17	76126851	76139049	7.03E-03
Marco	*MARCO*	2	119699742	119752236	8.90E-03
Cox7a1	*COX7A1*	19	36641824	36643771	9.05E-03
Lilra5, Lilra6, Pira2, Pirb, Gm14548, Gm15922, Gm15922	*LILRB4*	19	55155340	55181810	9.11E-03

p-value is not multiple testing corrected.
Key: AD, Alzheimer's disease.
Full network given in Supplementary Table 2.
