# Supplementary material for: Human longevity and Alzheimer’s disease variants act via microglia and oligodendrocyte gene networks
Source: Brain. 2025 Jan 9;148(3):969–84. doi: 10.1093/brain/awae339 (PMC11884759; doi:10.1093/brain/awae339)
Supplement: awae339_Supplementary_Data [file awae339_supplementary_data.zip › brain-2024-00123-File015.pdf]

Supplementary Table 5. Human hippocampus bulk RNA-seq oligodendrocytic module genes and their gene-based analysis p-value for Alzheimer's disease.

Mouse Symbol	Human Symbol	Human Chromosome	Start Location	End Location	AD Gene P-value
Clasrp	*CLASRP*	19	45542298	45574214	1.45E-13
Hnrnpa2b1	*HNRNPA2B1*	7	26212677	26241149	1.24E-04
Tex22	*TEX22*	14	105864916	105916443	3.00E-04
Zkscan1	*ZKSCAN1*	7	99613195	99639312	5.63E-04
Shc4	*SHC4*	15	49115932	49255641	9.78E-04
-	*BBIP1*	10	112658488	112679032	1.45E-03
Folh1	*FOLH1*	11	49168187	49230222	1.51E-03
Slc45a3	*SLC45A3*	1	205626979	205649587	1.77E-03
Cdr2l	*CDR2L*	17	72983727	73001895	1.80E-03
Fam76b	*FAM76B*	11	95502106	95523573	1.91E-03
Zfpl1	*ZFPL1*	11	64851682	64855872	1.99E-03
Pdcd4	*PDCD4*	10	112631553	112659764	2.08E-03
Tmem37	*TMEM37*	2	120187477	120196096	2.40E-03
Slc20a2	*SLC20A2*	8	42273993	42397069	2.50E-03
Pvrig	*PVRIG*	7	99815864	99819113	3.58E-03
Cenpc1	*CENPC*	4	68334466	68411324	3.78E-03
Tmed7	*TMED7*	5	114949205	114968689	3.98E-03
Slc26a9	*SLC26A9*	1	205882176	205912588	4.40E-03
Stag3	*STAG3*	7	99775186	99819111	4.59E-03
Acp7	*ACP7*	19	39574553	39602133	4.85E-03
Snx1	*SNX1*	15	64386322	64438289	4.85E-03
Tmem42	*TMEM42*	3	44903361	44907162	5.31E-03
Tardbp	*TARDBP*	1	11072401	11086477	5.59E-03
Unc5cl	*UNC5CL*	6	40994650	41006956	6.39E-03
Slf2	*SLF2*	10	102672326	102724893	6.64E-03
Mettl14	*METTL14*	4	119606523	119636588	7.43E-03
Fam222a	*FAM222A*	12	110152033	110208312	7.52E-03
Slc4a9	*SLC4A9*	5	139739787	139754728	8.65E-03
Uba6	*UBA6*	4	68478370	68566897	9.57E-03

p-value is not multiple testing corrected.
Key: AD, Alzheimer's disease.
Full network given in Supplementary Table 2.
