# Supplementary material for: Human longevity and Alzheimer’s disease variants act via microglia and oligodendrocyte gene networks
Source: Brain. 2025 Jan 9;148(3):969–84. doi: 10.1093/brain/awae339 (PMC11884759; doi:10.1093/brain/awae339)
Supplement: awae339_Supplementary_Data [file awae339_supplementary_data.zip › brain-2024-00123-File017.pdf]

Supplementary Table 7.1. Mouse hippocampus scRNA-seq activated response microglial, ARM, module genes and their gene-based analysis p-value for Alzheimer's disease.

Mouse Symbol	Human Symbol	Human Chromosome	Start Location	End Location	AD Gene P-value
Apoe	*APOE*	19	45409011	45412650	4.29E-81
Relb	*RELB*	19	45504688	45541456	6.56E-25
Ms4a6c	*MS4A6A*	11	59939487	59952139	2.02E-12
Pvr	*PVR*	19	45147098	45166850	1.73E-11
Ptk2b	*PTK2B*	8	27168999	27316908	3.58E-07
H2-Ab1	*HLA-DQB1*	6	32627244	32636160	2.92E-05
Plekha1	*PLEKHA1*	10	124134212	124202118	6.88E-04
Ydjc	*YDJC*	22	21982378	21984353	9.94E-04
Pirb	*LILRA5*	19	54818353	54824409	1.30E-03
Trim37	*TRIM37*	17	57059999	57184282	1.79E-03
Nrp1	*NRP1*	10	33466420	33625190	2.65E-03
Stbd1	*STBD1*	4	77227179	77232752	3.02E-03
Alkbh2	*ALKBH2*	12	109525993	109531436	4.03E-03
Ank	*ANKH*	5	14704909	14871894	6.30E-03
Tnip2	*TNIP2*	4	2743375	2758103	8.29E-03
Wdr55	*WDR55*	5	140044261	140053709	8.80E-03
Pirb	*LILRB4*	19	55155340	55181810	9.11E-03

p-value is not multiple testing corrected.
Key: AD, Alzheimer's disease.
Full network given in Supplementary Table 6.1.

Supplementary Table 7.2. Mouse hippocampus scRNA-seq phagolysosomal module genes and their gene-based analysis p-value for Alzheimer's disease.

Mouse Symbol	Human Symbol	Human Chromosome	Start Location	End Location	AD Gene P-value
Tomm40	*TOMM40*	19	45393826	45406946	3.07E-111
Clptm1	*CLPTM1*	19	45457842	45496599	7.46E-18
Ms4a6d	*MS4A6A*	11	59939487	59952139	2.02E-12
Spi1	*SPI1*	11	47376411	47400127	8.98E-12
Psmc3	*PSMC3*	11	47440320	47448024	1.15E-09
Trem2	*TREM2*	6	41126244	41130924	1.42E-08
Fbxo46	*FBXO46*	19	46213887	46234162	6.55E-08
Hbegf	*HBEGF*	5	139712428	139726216	3.88E-07
Gemin7	*GEMIN7*	19	45582453	45594782	1.85E-06
Isyna1	*ISYNA1*	19	18545198	18549111	1.34E-05
Pfdn1	*PFDN1*	5	139624624	139682706	4.12E-05
Ssbp4	*SSBP4*	19	18529674	18545372	4.91E-05
Mtch2	*MTCH2*	11	47638867	47664175	1.08E-04
Cnpy4	*CNPY4*	7	99717236	99723134	1.71E-04
Fcf1	*FCF1*	14	75179847	75205323	9.68E-04
Ndufaf6	*NDUFAF6*	8	95907995	96128683	1.08E-03
Lilra5	*LILRA5*	19	54818353	54824409	1.30E-03
Dlst	*DLST*	14	75348594	75370448	1.39E-03
Actb	*ACTB*	7	5566778	5603415	1.64E-03
Csnk2b	*CSNK2B*	6	31633013	31638120	1.76E-03
Arpc1a	*ARPC1A*	7	98923533	98963885	1.76E-03
Zyx	*ZYX*	7	143078173	143088204	2.00E-03
Prkra	*PRKRA*	2	179296141	179316239	2.10E-03
Tmem37	*TMEM37*	2	120187477	120196096	2.40E-03
Zfp655	*ZNF655*	7	99156029	99174076	2.80E-03
Pkp4	*PKP4*	2	159313476	159539391	2.97E-03
Psmc6	*PSMC6*	14	53173890	53195305	3.74E-03
Fam220a	*FAM220A*	7	6369040	6388612	4.78E-03
Snx1	*SNX1*	15	64386322	64438289	4.85E-03
Tmem42	*TMEM42*	3	44903361	44907162	5.31E-03
Bnip3l	*BNIP3L*	8	26240414	26363152	5.42E-03
Ddah2	*DDAH2*	6	31694815	31698394	5.98E-03
Mrpl43	*MRPL43*	10	102729215	102747272	6.03E-03
Eif1b	*EIF1B*	3	40351175	40353915	6.89E-03
2900026A02Rik	*KIAA1671*	22	25348697	25593415	7.14E-03
Zmat2	*ZMAT2*	5	140078265	140086261	7.57E-03
Polr2e	*POLR2E*	19	1086578	1095379	7.88E-03
Ech1	*ECH1*	19	39306062	39322645	8.23E-03
Ik	*IK*	5	140026643	140042064	8.81E-03
Lilra5	*LILRB4*	19	55155340	55181810	9.11E-03
Oser1	*OSER1*	20	42825136	42839431	9.42E-03
Etf1	*ETF1*	5	137841784	137878989	9.58E-03
Skp1a	*SKP1*	5	133484626	133512729	9.81E-03
Mrpl58	*MRPL58*	17	73008765	73017356	9.92E-03
Grn	*GRN*	17	42422454	42430470	1.00E-02

p-value is not multiple testing corrected.
Key: AD, Alzheimer's disease.
Full network given in Supplementary Table 6.4.

Supplementary Table 7.3. Mouse hippocampus scRNA-seq homeostatic microglial subcluster 2, HM2, module genes and their gene-based analysis p-value for longevity.

Mouse Symbol	Human Symbol	Human Chromosome	Start Location	End Location	Longevity Gene P-value
Fes	*FES*	15	91426925	91439006	1.59E-07
Zkscan5	*ZKSCAN5*	7	99102274	99132323	1.13E-05
Ptcd1	*PTCD1*	7	99014362	99063786	3.28E-05
Ptcd1	*ATP5MF-PTCD1*	7	99017372	99063820	3.59E-05
Jam3	*JAM3*	11	133938820	134021896	1.77E-04
Casp8	*CASP8*	2	202098166	202152434	2.80E-04
Mafk	*MAFK*	7	1570350	1582679	5.51E-04
Mcrs1	*MCRS1*	12	49950327	49961936	9.35E-04
Golph3l	*GOLPH3L*	1	150618701	150669620	1.47E-03
Rbm4	*RBM4*	11	66406088	66435845	1.56E-03
Ppp2r3c	*PPP2R3C*	14	35554673	35591723	1.62E-03
Bcap29	*BCAP29*	7	107220422	107269615	1.66E-03
Rit1	*RIT1*	1	155867599	155881195	1.86E-03
Srp54b	*SRP54*	14	35451163	35498773	1.92E-03
1700066M21Rik	*C2orf69*	2	200775979	200820658	1.96E-03
Cln8	*CLN8*	8	1703944	1749877	2.05E-03
Plpp3	*PLPP3*	1	56960419	57110974	2.58E-03
Maip1	*MAIP1*	2	200820040	200873263	3.01E-03
Bud31	*BUD31*	7	99006264	99017239	3.22E-03
Stat3	*STAT3*	17	40465342	40540586	3.25E-03
Scamp5	*SCAMP5*	15	75249560	75313837	3.51E-03
Klc2	*KLC2*	11	66024765	66035331	3.70E-03
Ptpn1	*PTPN1*	20	49126858	49201778	4.24E-03
Zkscan14	*ZNF394*	7	99084142	99097947	5.02E-03
Zfp664	*ZNF664*	12	124456392	124499986	5.63E-03
Exoc3	*EXOC3*	5	443273	472052	5.66E-03
Dusp6	*DUSP6*	12	89741009	89747048	5.76E-03
Slc44a2	*SLC44A2*	19	10713133	10755235	6.45E-03
Xpc	*XPC*	3	14186647	14220283	6.56E-03
Tap2	*TAP2*	6	32789610	32806557	6.57E-03
Ctsf	*CTSF*	11	66330934	66336312	6.69E-03
Csnk1a1	*CSNK1A1*	5	148871760	148931115	6.84E-03
Gstz1	*GSTZ1*	14	77787227	77797940	7.66E-03
Fads1	*FADS1*	11	61567097	61596790	7.85E-03
Usp38	*USP38*	4	144106070	144144983	8.63E-03
F11r	*F11R*	1	160965001	160991138	9.06E-03
Bsn	*BSN*	3	49591922	49708978	9.08E-03
Abhd16a	*ABHD16A*	6	31654726	31671221	9.35E-03
Zscan29	*ZSCAN29*	15	43650370	43663223	9.44E-03

p-value is not multiple testing corrected.
Full network given in Supplementary Table 6.3.
